# Svep1 orchestrates distal airway patterning and alveolar differentiation in murine lung development

**DOI:** 10.7554/eLife.100443

**Published:** 2026-10-12

**Authors:** Nicole Foxworth, Julie Wells, Sara Ocana-Lopez, S Muller, Pooja Bhayani, James Denegre, Kristina Palmer, Wendy Memishian, Teresa McGee, Stephen A Murray, Patricia K Donahoe, Carol J Bult, Maria Loscertales

**Affiliations:** 1 Pediatric Surgical Research Laboratories, Massachusetts General Hospital Boston United States; 2 The Jackson Laboratory Bar Harbor United States; 3 Department of Surgery, Harvard Medical School Boston United States; Ashoka University India; Universitat de Barcelona Spain

**Keywords:** lung morphogenesis, airway architecture, branching patterning, alveolarization, cell progenitor, alveolar cell differentiation, Mouse

## Abstract

Disruptions in airway branching or alveolar differentiation during lung development can lead to severe respiratory deficiencies and neonatal death. The molecular mechanisms governing branching patterning and early alveolar formation remain elusive. Loss of *Svep1* function in mice results in various developmental defects, including lung hypoplasia and perinatal lethality. Our examination of the lungs of *Svep1* knockout (*Svep1^-/-^*) mouse embryos, both in vivo and in vitro, revealed that *Svep1* mutants exhibit an increase in the number of disorganized distal airway tips, progressively greater disruption of lung lobe morphology over time, and disrupted saccular development. *Svep1* interacts with FGF signaling to regulate smooth muscle differentiation and, together with *Fgf9*, guides airway branching patterning. Transcriptomic data from the lungs of *Svep1^-/-^* embryos revealed dysregulated gene expression affecting saccular maturation. Our findings demonstrate that *Svep1* is a key extracellular matrix player shaping airway morphology and influencing alveolar fate. These insights offer potential avenues for therapeutic interventions in congenital lung disorders.

## Introduction

The architectural blueprint of the lungs emerges from two fundamental developmental processes: branching morphogenesis, responsible for constructing the intricate airway tree structure, and differentiation, which generates specialized cells in distinct lung regions, predominantly after the formation of the tracheobronchial tree (Rawlins, 2011). The cessation of molecular genetic signaling promoting branching is a prerequisite for distal alveolar cell differentiation (Chang et al., 2013; Frank et al., 2019).

In mice, lung development begins at embryonic day 9.5 (E9.5) (Ten Have-Opbroek, 1991; Warburton et al., 2010) when two primary buds emerge from the ventral foregut. During the pseudoglandular stage (E12.5–16.5), epithelial progenitors at the bud tips undergo an iterative process of budding and bifurcation into the surrounding mesenchyme, and this is followed by expansion of the diameter and length of the respiratory tree during the canalicular stage (E16.5–17.5). During the saccular stage (E17.5–P5), the vasculature develops along the airway and terminal airway tips widen. The alveolar epithelium subsequently differentiates into alveolar type 2 (AT2) cells, responsible for surfactant protein C (SFPC) production, and the thin, gas-exchanging alveolar type 1 (AT1) cells. Primitive septa arise at this stage. Secondary septation and alveolar maturation occur during the postnatal alveolar stage (P5–30) (Warburton et al., 2010).

The geometry of the airway tree is generated by three local branching modes: domain, planar, and orthogonal (Metzger et al., 2008). During domain branching, new branches grow perpendicularly around the circumference of the parent branch and generate the initial scaffold of each lobe and the overall shape of the lung. Planar bifurcation occurs at the tips of the airway epithelium and generates branches in the same plane, resulting in the thin edges of the lung lobes. Orthogonal bifurcation generates branches perpendicular to prior branches, thus filling in the interior of the lung. Trifurcations are rare and appear principally at the lung surface (Blanc et al., 2012; Zhao et al., 2009). As branching proceeds, proximal-distal patterning is established, and cellular specification follows Frank et al., 2019; Rawlins et al., 2009; Yang et al., 2018; Zhang et al., 2024. Lung bud tip progenitors persist and proliferate and subsequently undergo differentiation into AT1 and AT2 cells (Frank et al., 2019). The cells of the primal airway stalk differentiate into multiple proximal cell types (Conway et al., 2019; Rock and Hogan, 2011; Herriges and Morrisey, 2014; Whitsett et al., 2019).

Studies in laboratory mice have shown that branching morphogenesis requires mesenchymal-epithelial interactions, and various signaling pathways, particularly the FGF pathway, play important roles in branching (Morrisey and Hogan, 2010). Fibroblast growth factor 10 (FGF10) is secreted by mesenchymal cells and acts via the *FGFR2* receptor to maintain a permissive environment for branching morphogenesis. FGF10 maintains distal epithelial SOX9^+^ progenitor cells in an undifferentiated state. These cells play a pivotal role in subsequent alveolar and mesenchymal differentiation (Bellusci et al., 1997; Min et al., 1998; Sekine et al., 1999; De Moerlooze et al., 2000; Weaver et al., 2000; Abler et al., 2009; Ramasamy et al., 2007; Warburton, 2008; Hyatt et al., 2004; Ornitz and Yin, 2012; Volckaert et al., 2013; Park et al., 1998). FGF10^+^ cells give rise to bronchial airway smooth muscle cells during early branching (Warburton et al., 2005; Li et al., 2018; Mailleux et al., 2005; Yuan et al., 2018). FGF9 is also required for lung development and plays different roles depending on its spatial and temporal expression. In the mesothelium, FGF9 plays a primary role in mesenchymal growth; in the epithelium, FGF9 controls epithelial branching (Colvin et al., 1999; Colvin et al., 2001; del Moral et al., 2006; Yin et al., 2011). The extracellular matrix (ECM) also plays important roles in epithelial branching and alveolarization (Zhou et al., 2018; Mecham, 2018; Nguyen et al., 2002; Sakai et al., 2003; Chen and Krasnow, 2012; Loscertales et al., 2016) and contributes to organ shape (Walma and Yamada, 2020).

*Svep1* (Sushi, von Willebrand factor type A, EGF, and pentraxin domain-containing 1), also known as Polydom, encodes an ECM protein that is ubiquitously expressed in the tissues of adult mice and plays roles in cell adhesion (Sato-Nishiuchi et al., 2012), epidermal differentiation (Samuelov et al., 2017), and lymphatic vessel formation and remodeling (Karpanen et al., 2017; Morooka et al., 2017). *Svep1* homozygous mutant mice die at birth with edema and body abnormalities (Samuelov et al., 2017; Morooka et al., 2017; Kishore et al., 2020; Dickinson et al., 2016). We investigated the role of *Svep1* in the development of lungs of mice homozygous for the *Svep1^tm1b(EUCOMM)Hmg^* allele (hereafter, *Svep1^-/-^*) generated by the Knockout Mouse Phenotyping Program (KOMP2) (Dickinson et al., 2016). We observed that the loss of *Svep1* disrupts the basic lung branching program, resulting in irregular lung lobe shape due to an aberrant increase in distal airway tips. Smooth muscle differentiation is also disrupted in the lungs of *Svep1^-/-^* embryos. Through the treatment of lung explant cultures with exogenous SVEP1 protein, we demonstrated that *Svep1* functions as an inhibitor of branching morphogenesis. Furthermore, we demonstrated that inactivation of FGF signaling recovers smooth muscle differentiation in *Svep1^-/-^* lungs and that exogenous FGF9 treatment restores SVEP1 branching inhibition, implying a potential role for SVEP1-FGF9 signaling specific to branching.

## Results

### Svep1 is expressed in lung mesenchyme with high expression at the lung periphery

**Figure 1.**
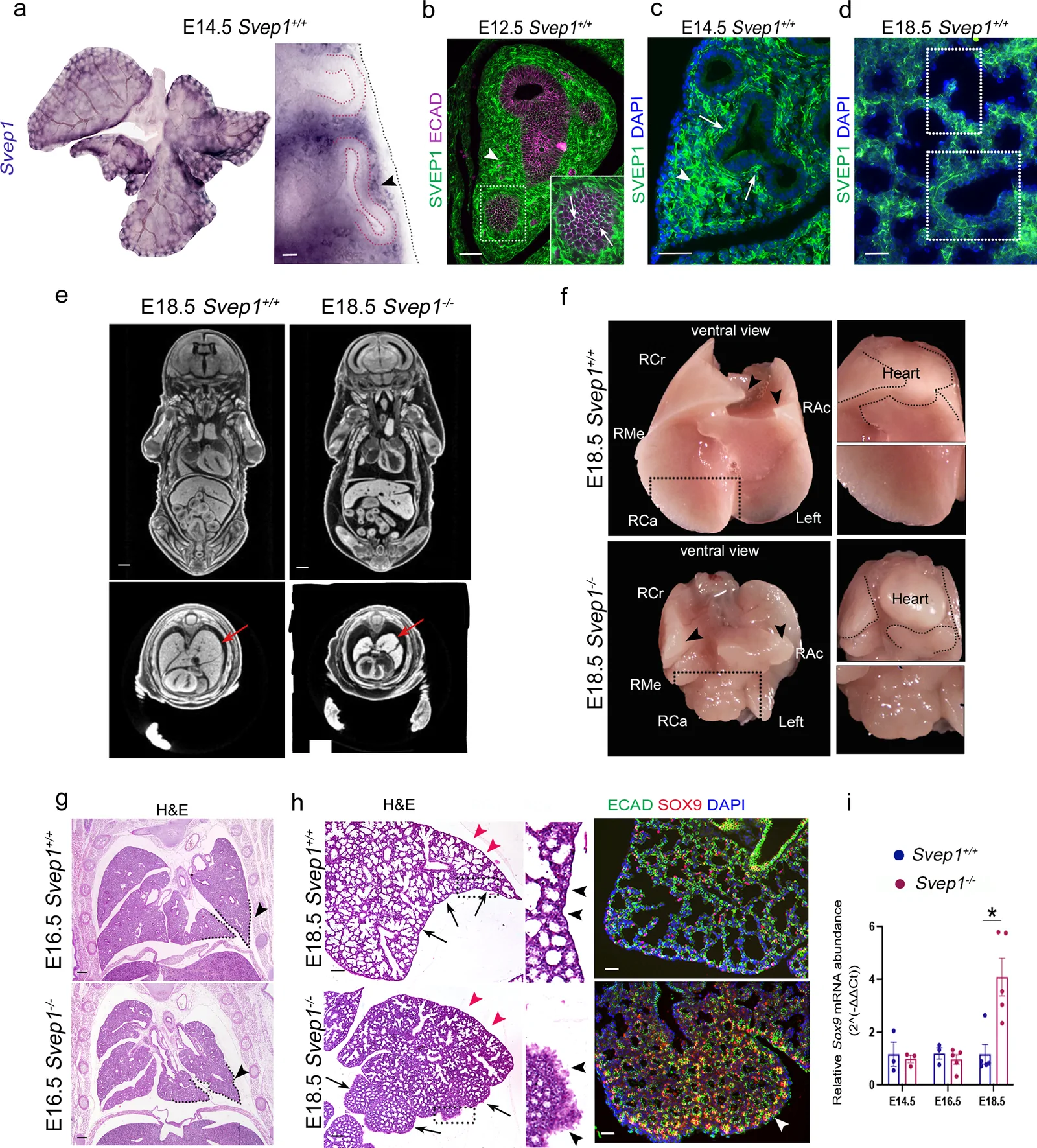
Svep1 expression pattern and Svep1^-/-^ hypoplastic lung lobes anomalies. (**a**) In situ hybridization of lungs from embryonic day 14.5 (E14.5) wild-type embryos showed strong *Svep1* mRNA expression in the mesenchyme at the lung periphery and around bifurcating distal airway tips (right inset outlined in red, arrowhead). (**b**) Arrowhead shows mesenchymal expression of SVEP1 protein in normal E12.5 lung. White arrows indicate the location of the SVEP1 protein on the epithelial membrane stained with ECAD (purple) in a distal airway bud. (**c**) SVEP1 expression is strong in the mesenchyme toward the lobe tip of the E14.5 lungs and branching cleft sites (arrowheads). (**d**) SVEP1 protein localizes in the lung parenchyma, including the primary septa (upper box) and adjacent to the proximal airway epithelium (lower box). (**e**) microCT scan of E18.5 embryos showing multiple defects, including thinning of the diaphragm and lung hypoplasia (red arrows). (**f**) Ventral view of E18.5 of *Svep1^+/+^* and *Svep1^‐/-^* lungs. The arrowheads indicate the tips of the lung lobes, which are short and rounded (highlight in black) in *Svep1^-/-^* mice and have a cauliflower morphology. (**g**) Hematoxylin and eosin (H&E)-stained lung section embryos demonstrate that E16.5 lungs from *Svep1^-/-^* embryos are smaller and have irregular lobe edges. Black arrowheads indicate left lobes with highlighted surface. (**h**) The red arrowheads show the dorsal side of the E18 lungs, whereas the black arrows highlight the ventral side. *Svep1^-/-^* embryos show defective saccular development mainly at the edge on the ventral side of the lung (inserts arrowheads). Co-localization of ECAD and SOX9 confirms high expression of SOX9 in distal airways (white arrowheads) in the lung of *Svep1^-/-^* embryos at E18.5. (**i**) Plots showing *Sox9* relative mRNA expression at E14.5, E16.5, and E18.5. mRNA expression is significantly higher at E18.5 in *Svep1^-/-^* embryos (mean ± SEM; Wilcoxon rank-sum test; p<0.05). Right cranial (RCr), right caudal (RCa), right medial (RMe), and right accessory (RAc) lobes. Scale bars: 50 μm (**a–d**), 100 μm. Figure 1—source data 1.qPCR data for Sox9 expression at E14.5, E16.5, and E18.5 (Figure 1i).

*Svep1* is expressed in the lung mesenchyme, especially at the tip and edges of the lung (Figure 1a–d, Figure 1—figure supplement 1a–b). *Svep*1 mRNA expression was strong in the mesenchyme adjacent to the distal epithelial airways and around bifurcating tips (Figure 1a, Figure 1—figure supplement 1a). We detected SVEP1 protein expression on the epithelium membrane (ECAD+) of very distal lung buds (Figure 1b, Figure 1—figure supplement 1b) and microvasculature (Figure 1—figure supplement 1). At the saccular stage (E18.5), transcript and protein expression of *Svep1* was detected in the primary septa of lung parenchyma and in the mesenchyme next to the proximal epithelium (Figure 1d, Figure 1—figure supplement 1d). Consistent with previous findings, *Svep1* expression during lung development was high between E14.5 and E18.5 (Beauchemin et al., 2016; Figure 1—figure supplement 1e). Single-cell RNA sequencing (scRNA-seq) data demonstrated that expression of *Svep1* in the lungs of E18.5 wild-type embryos was prominent in matrix fibroblasts (Figure 5—figure supplement 1a).

**Figure 1—figure supplement 1.**
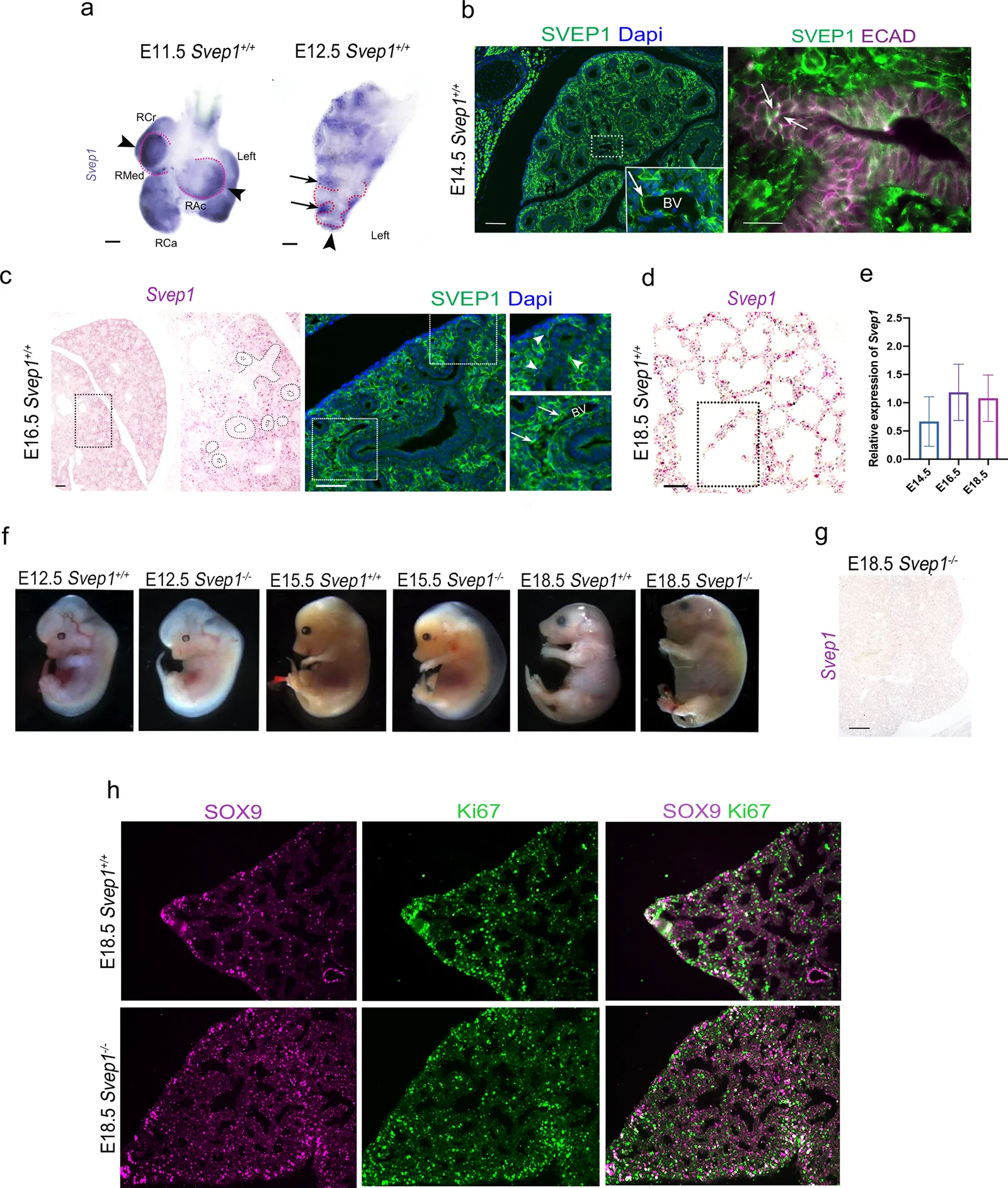
Expression of Svep1 in the developing lung of wild-type C57BL/6J mouse embryos. (**a–b**) Svep1 in situ hybridization in embryonic day 11.5 (E11.5) and E12.5 embryos. Arrowheads indicate high expression of Svep1 at the distal tip of right medial (RMe) and accessory (RAc) lobes (outlined in red) and the left lobe. Arrows show Svep1 expression along and between early airway buds (outlined in red). (**b**) Arrows mark the expression of SVEP1 (green) adjacent to epithelial cells (ECAD; purple) in E14.5 normal lung. (**c**) In situ hybridization showing Svep1 expression in wild-type E16.5 lungs. The area outlined in the left panel is shown at higher magnification in the right-hand panel to highlight airways. Svep1 expression is stronger at the edges of the lung (arrowheads). SVEP1 protein localized next to distal airways (arrowheads) and microvasculature (arrows). (**d**) Svep1 protein or mRNA is seen in the mesenchyme at E18.5 and is highlighted in the primary septum. (**e**) RT-qPCR for Svep1 expression showing relative fold change at E14.5, E16.5, and E18.5. (**f**) Normal and Svep1^-/-^ embryos at E12.5, E15.5, and E18.5 show edema and cranial, limb, and tail developmental defects increasing in mutants over time. (**g**) Svep1 mRNA is absent in E18.5 mutants. (**h**) SOX9 and KI67 mark proliferating epithelial progenitor cells in E18.5 lungs, mainly at the lung periphery in Svep1^-/-^ mutants. Scale bars: 100 µm (**a**, **c**, **d**, **h**); 200 µm (**b**). Figure 1—figure supplement 1—source data 1.qPCR expression data for Svep1 at E14.5, E16.5, E18.5 (Figure 1—figure supplement 1e).

### Hypoplastic and aberrant lung shape in Svep1^-/-^ embryos

Consistent with previous reports (Dickinson et al., 2016), *Svep1^-/-^* embryos displayed perinatal lethality, whole-body edema, and lung hypoplasia that was prominent at E18.5 by microCT (Figure 1e and Figure 1—figure supplement 1). In situ hybridization (ISH) confirmed the absence of *Svep1* expression in the lungs of homozygous knockout animals (Figure 1—figure supplement 1g). Examination of gross examination of embryos at E18.5 revealed small lungs with lobes that failed to enclose the heart and had a highly lobulated, cauliflower-like appearance (Figure 1f). Histological analysis at E16.5 showed small lungs in mutants with irregular shape; at E18.5, small saccules were observed along a dorsal-ventral gradient, accompanied by a reduced lumen primarily on the ventral side (Figure 1g and h). Noticeable differences, including a thickening along the lobulated ventral edges of the lung composed of mesothelium, were observed in *Svep1^-/-^* embryos compared to wild-type embryos, reflecting developmental abnormalities in the lung mesothelium of mutant mice (Figure 1h).

### Saccular development defects in Svep1^-/-^ embryos

Sacculation begins in the murine lung shortly after E16.5, with the flattening of AT1 cells at the proximal-to-distal opening of the distal airway lumen. Cells in the developing distal airways at the lung edge are the last to differentiate (Zhang et al., 2024; Li et al., 2018; Desai et al., 2014). Examination of distal airway morphology of *Svep1^-/-^* embryos at E18.5, through labeling the lung epithelium with ECAD and the distal progenitor marker SRY-box transcription factor 9 (SOX9), revealed that the densely packed distal airways at the ventral edge exhibited a morphology typical of the earlier pseudoglandular stage instead of the saccular stage (Figure 1h). Quantitative real-time PCR (qPCR) confirmed elevated expression of *Sox9* in the lungs of *Svep1* knockout embryos at E18.5 but not at earlier time points (Figure 1i). Co-staining of SOX9 with the cell proliferation marker KI67 showed proliferating epithelial progenitor cells in E18.5 lungs, mainly at the lung periphery in *Svep1^-/-^* mutants (Figure 1—figure supplement 1h). Our results indicate that the distal epithelium in *Svep1* lungs is in an earlier developmental stage, characterized by a higher proportion of epithelial cells in a progenitor stage, rather than in the saccular period when cells are differentiating into specialized lung epithelial type I and II cells. Our finding highlights the essential role of Svep1 in the maturation of the distal lung epithelium.

### Lungs of Svep1^-/-^ embryos show an aberrant increase in distal airways, altering lung lobe shape

**Figure 2.**
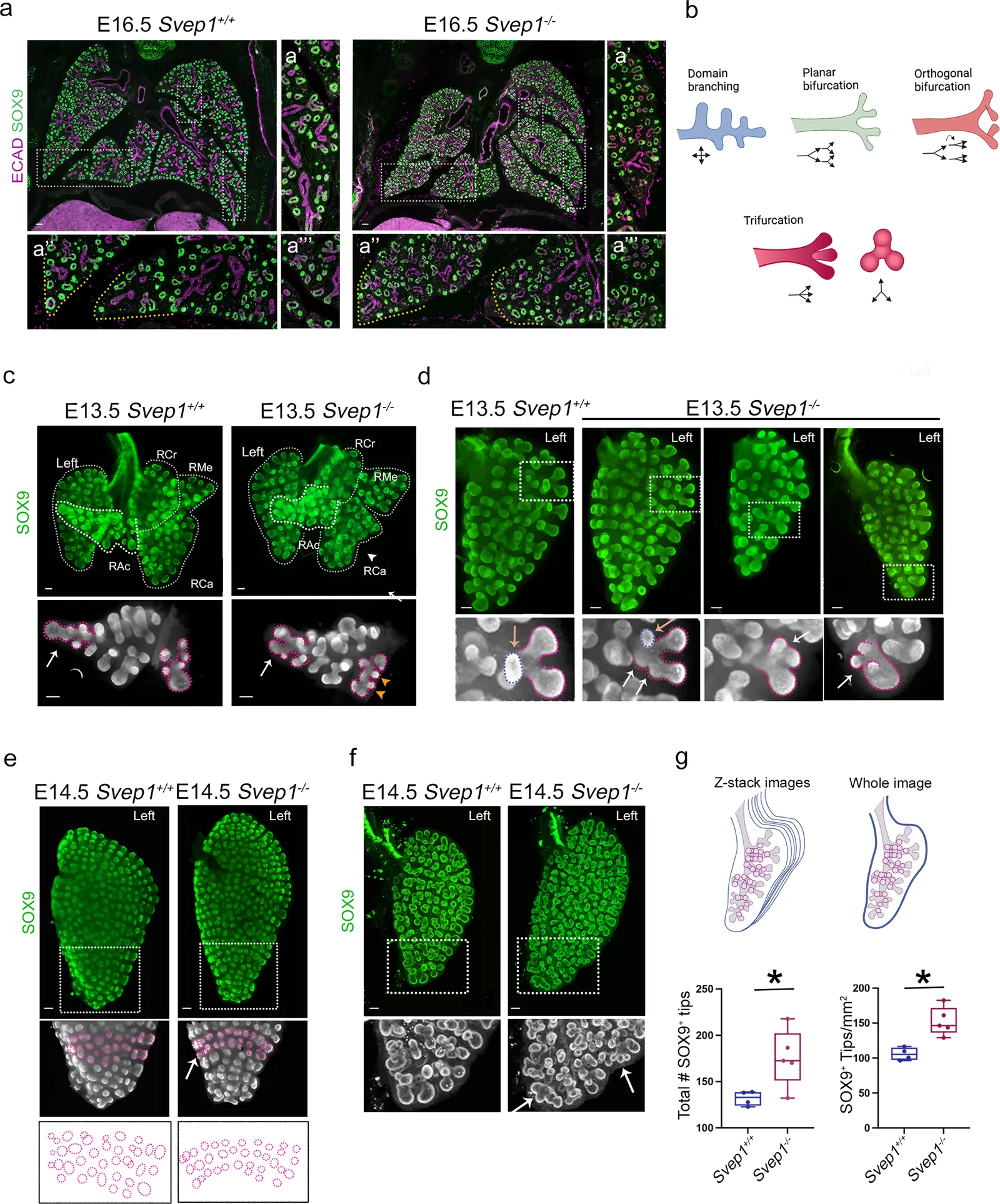
Airway organization defects and abnormal branching patterns in *Svep1^-/-^* embryonic lungs resulting in lung lobe shape anomalies. (**a**) Co-localization of epithelial (ECAD) and epithelial progenitor (SOX9) markers showing distal airways in lungs of embryonic day 16.5 (E16.5) mice. (**a'**) The blue highlights in inserts represent the distal tip of the normal and mutant RAc lung lobes, with the mutant left lobe showing multiple branching. (**a''**) Inserts illustrate airway density at the lung periphery in wild-type (WT) and mutants, with mutants having higher density. (**a'''**) In *Svep1^-/-^* lungs, distal airway tips are organized in packed airway rosettes on the ventral side of the lung lobe. (**b**) Illustration of the three branching modes that pattern the airways of the lung (domain branching, planar bifurcation, orthogonal bifurcation) and distal trifurcation. This panel was created using BioRender.com. (**c–f**) Representative Z-stack images show SOX9 immunofluorescence. (**c**) E13.5 whole lungs of normal and *Svep1^-/-^* embryos. The images show *Svep1^-/-^* lungs with abnormal RCa lobe morphology (arrowheads), RAc lobe distal tip branching (arrows), and secondary branching patterning (orange arrowheads). (**d**) Pictures of E13.5 *Svep1^+/+^* and *Svep1^-/-^* embryos showing ectopic branching/budding anomalies in mutant embryos (areas outlined in pink in lower inserts). (**e**) E14.5 left lung lobes of *Svep1^+/+^* and *Svep1^‐/‐^* embryos. SOX9^+^ buds highlighted in pink and cartoon show airway tip arrangement in normal and mutants. Arrow shows defective tip arrangement resulting in abnormal lung tip morphology in *Svep1^-/-^* embryos. (**f**) Cleared tissue immunofluorescence of SOX9 in the left lung lobe at E14.5 reveals airway disorganization in *Svep1^-/-^* embryos vs *Svep1^+/+^* embryos. Distal lung tips highlighted show branching trifurcations in the *Svep1^-/-^* embryo (white arrow) and aberrant distal tip airway. (**g**) Representation of the left lung lobe Z-stack for distal airway quantification. Whole lungs of *Svep1^-/-^* embryos at E14.5 have a statistically significant increase in the total number of SOX9 positive airway tips and ratio of tip and area compared to wild-type embryos (mean ± SEM; two-tailed unpaired t-test; p<0.05). This panel was created using BioRender.com. Right cranial (RCr), right caudal (RCa), right medial (RMe), and right accessory (RAc) lobes. Scale bars: 100 μm (**a–e**). Figure 2—source data 1.Counts of Sox9 positive airway tips in lungs from wild type and mutant Svep1 embryos at E14.5 (Figure 2g).

To determine whether *Svep1^-/-^* embryos have branching defects, we stained E16.5 lungs with SOX9 and ECAD, which revealed densely packed distal airways in *Svep1^-/-^* embryos. Mutant lobe tips had multiple distal branches and rosettes on the ventral lobe side (Figure 2a). To further examine defects in the branching program modes (Metzger et al., 2008; Figure 2b) in *Svep1* mutant lungs, we stained whole lung and individual lobes with SOX9 at E13.5, E14.5, and E15.5 and showed that *Svep1* branching defects accumulate over time (Figure 2c–e and Figure S2a,b). We observed branching and lobe shape anomalies in *Svep1^-/-^* lungs as early as E13.5, primarily at the lung tips and edges (Figure 2c and d). Moreover, the branching abnormalities observed in the mutants were not uniform across the lung lobes, leading to different outcomes in the shape of the lobes. At E13.5, for example, the shape of the right caudal (RCa) lobe was narrower in *Svep1^-/-^* embryos compared to wild-type embryos. The right accessory (RAc) and right medial (RMe) lobes of *Svep1* mutant embryos terminated in two or more buds instead of a single tip typically observed in lungs of wild-type embryos (Figure 2c). Further examination of the left lobes showed that *Svep1^-/-^* embryos at E13.5 exhibited peripheral branch mis-localization and additional budding, disrupting the normal planar bifurcation of the lung (Figure 2d, Figure 2—figure supplement 1a). When planar bifurcation is disrupted at the lobe tip, ectopic branching angles occur at the lung tips in mutants (Figure 2d). This observation suggests that *Svep1* deficiency impairs lung branching angles early in development.

**Figure 2—figure supplement 1.**
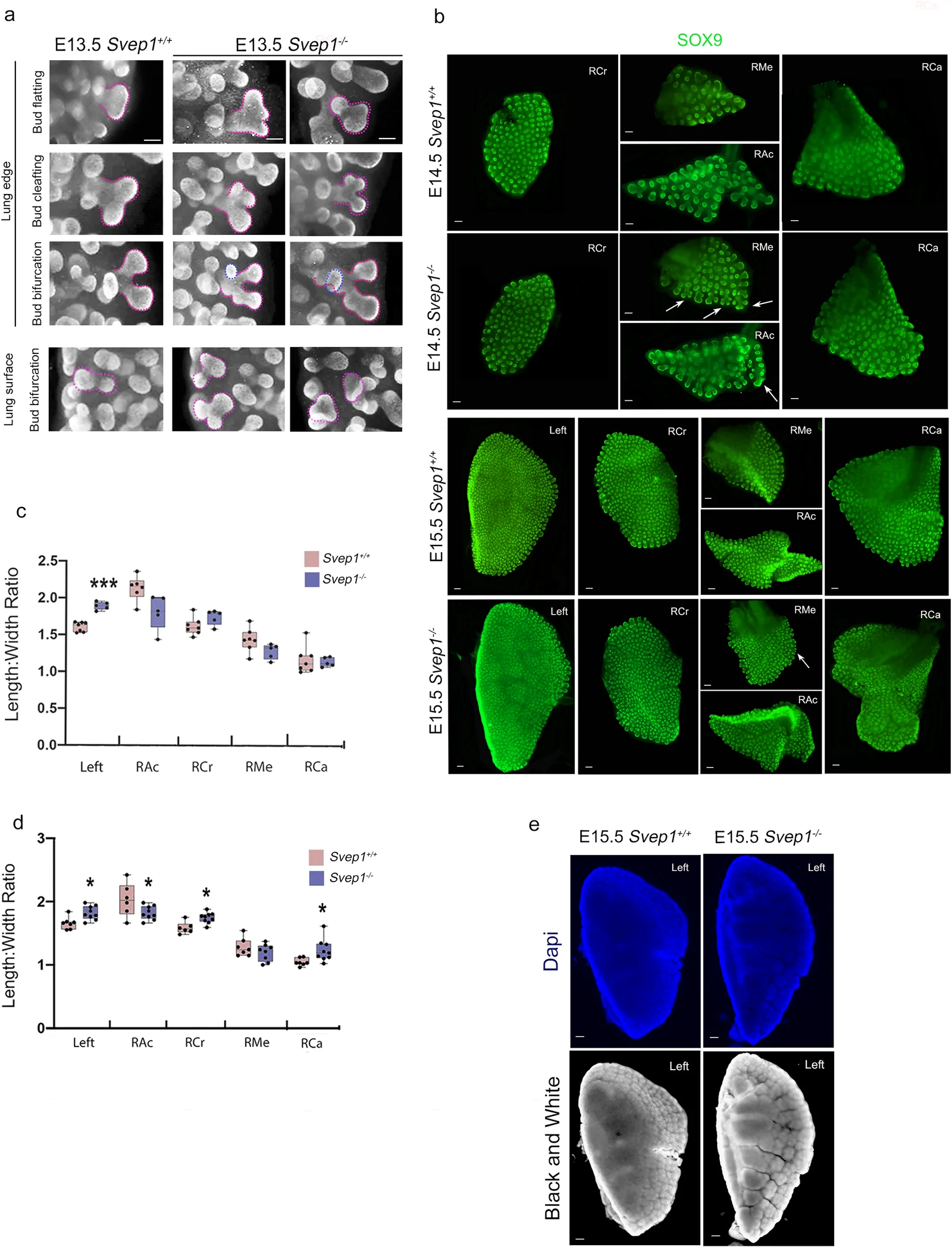
SOX9 whole-mount immunohistochemistry in lung lobes. (**a**) SOX9 immunostaining of embryonic day 13.5 (E13.5) lung lobe edges highlights (pink) Svep1 branching and bifurcation anomalies at the lung periphery. The blue highlights denote branching domain buds. (**b**) Immunofluorescence images of E14.5 and E15.5 lung lobes showing distal airways in both wild-type and Svep1^-/-^ embryos. Arrows indicate defective branching in Svep1^-/-^ embryos. (**c**) Graph of the ratio of length to width for individual E14.5 lobes. Svep1^+/+^ in pink; Svep1^-/-^ in blue. (**d**) Graph of the ratio of length to width for individual E15.5 lobes. Svep1^+/+^ in pink; Svep1^-/-^ in blue. (**e**) DAPI staining of E15.5 lung lobes displays the gross morphology of the lung surface for both Svep1^+/+^ and Svep1^-/-^ specimens. Average lobe length-to-width ratio ± SEM; p<0.01; n≥5. Lobe abbreviations: right cranial (RCr), right caudal (RCa), right medial (RMe), and right accessory (RAc) lobes. Scale bars: 100 µm. Figure 2—figure supplement 1—source data 1.Ratio of length to width in lungs Svep1 wild type and mutant embryos at E14.5 (Figure 2—figure supplement 1c). Figure 2—figure supplement 1—source data 2.Ratio of length to width in lungs Svep1 wild type and mutant embryos at E15.5 (Figure 2—figure supplement 1d).

The orthogonal bifurcation mode of the branching program fills the lung’s exterior and interior surfaces in a specific pattern where each new bud maintains its boundaries with others and creates the surface of the lung lobes (Metzger et al., 2008). However, this orthogonal pattern was disrupted in mutant embryos at E14.5, with the airways rearranged into a ‘row of beads’ pattern, resulting in abnormal rounded tips (Figure 2e). Moreover, the left lung lobes of E14.5 mutant embryos were 8% more elongated than those of wild-type *Svep1* embryos (length/width ratio [mm] *Svep1^+/+^*=1.60; *Svep1^-/-^*=1.90, p<0.001, t-test; Figure 2—figure supplement 1c). Tissue-cleared left lobes stained with SOX9 at E14.5 confirmed abnormal mutant left lobe morphology as well as multi-budding of the distal airways and peripheral trifurcations (Figure 2f).

At E15.5, aberrations persist in lung branching patterns along the margins and ventral side, leading to substantial alterations in lobe elongation in *Svep1^-/-^* embryos (Figure 2—figure supplement 1b, c, and d). Staining left lung lobes of *Svep1^-/-^* embryos at E15.5 with DAPI (4',6-diamidino-2-phenylindole) revealed prominent abnormalities on the lung surface (Figure 2—figure supplement 1e).

Quantification of SOX9+ in a whole lung Z-stack imaging revealed a significant increase in both the total number of SOX9+ distal tips and the ratio of tips to area by E14.5 (Figure 2g; p<0.05, t-test). Overall, these findings demonstrate that *Svep1* is crucial for normal branching morphogenesis, and its absence leads to disorganization of airway patterning, primarily affecting the tips, edges, and ventral areas of the lung, which in turn alters the overall shape of the lung lobes.

### Lungs of Svep1^-/-^ embryos have ectopic branching, decreased planar bifurcations, and increased trifurcations

**Figure 3.**
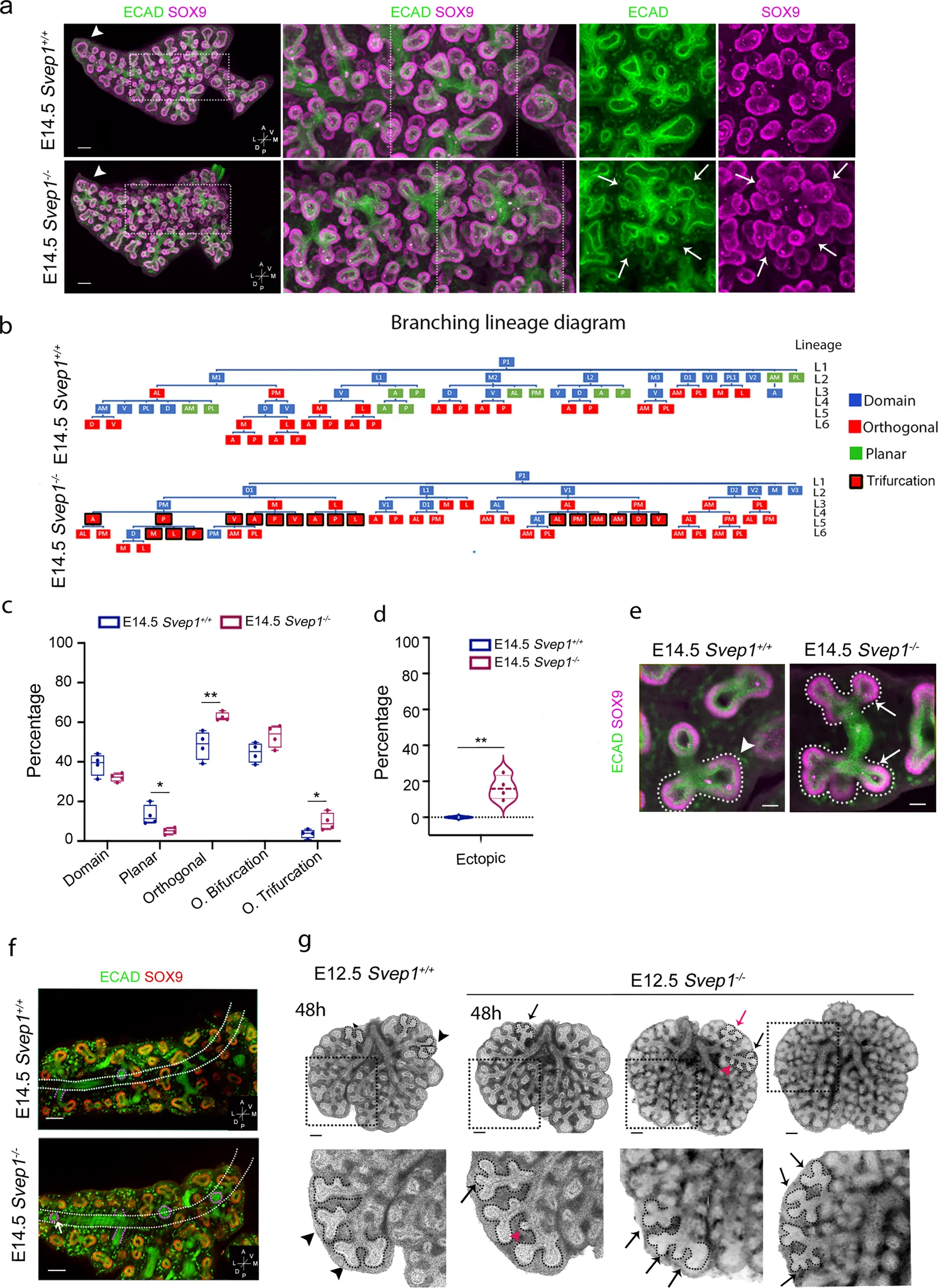
Disrupted branching programs and altered airway architecture in *Svep1^-/-^* right accessory (RAc) lung lobes at embryonic day 14.5 (E14.5). (**a**) Compilation of a Z-stack image series for tissue-cleared whole RAc lung lobes labeled with ECAD (green) and SOX9 (purple) highlights disorganized branching in *Svep1^-/-^* embryos at E14.5. Arrows (lower right) point to branching airways with rosette-like arrangements and ectopic airway tips growing in random directions. Arrowheads (left panels) at the right caudal (RCa) lobe tips indicate a single tip in the lobe of *Svep1^+/+^* embryos but multi-branched tips in *Svep1* knockout embryos. (**b**) Schematic illustrating representative domain (blue), planar (green), and orthogonal (red) branching program types of *Svep1^+/+^* and *Svep1^-/-^* RAc lobes. Lungs of *Svep1^-/-^* embryos lack planar bifurcations and have many trifurcating branch tips (red highlighted in black). (**c–d**) Graphs displaying the percentages of branching modes, orthogonal bifurcations, and trifurcations (**c**) and ectopic budding (**d**) in E14.5 RAc lobes of wild-type and *Svep1^-/-^* embryos. (**e**) Representative single Z-stack images of RAc lobes at E14.5, stained with ECAD (green) and SOX9 (purple), show bifurcations (white dotted lines and white arrowhead) in *Svep1^+/+^* embryos, but trifurcations (white dotted lines and white arrows) in *Svep1^-/-^* mice. (**f**) Single Z-stack image of RAc lobes stained with ECAD (green) and SOX9 (red) displays ectopic domain branching in *Svep1^-/-^* mice (arrow). (**g**) Lung explants from E12.5 embryos after 48 hr in organ culture. Explants from *Svep1^+/+^* embryos show normal bifurcation (black arrowheads), whereas explants from *Svep1^-/-^* embryos show trifurcations (black arrows) and ectopic budding (red arrowheads) (data are presented as mean ± SEM; two-tailed unpaired t-test; n=4 per genotype) (**c**, **d**). Scale bars: 100 μm (**a**, **g**); 50 μm *(**e***). Figure 3—source data 1.Percentages of branching modes, orthogonal bifurcations, and trifurcations in right accessor lung lobs of Svep1 wild type and mutant embryo at E14.5 (Figure 3c). Figure 3—source data 2.Percentages of ectopic budding in right accessory lung lobes of Svep1 wild type and mutant embryos at E14.5 (Figure 3d).

To investigate which of the main branching programs are affected in *Svep1* mutants, we modeled the branching patterns of normal and mutant lungs. We labeled tissue-cleared whole lungs from E14.5 embryos with ECAD and SOX9 and generated a 3D reconstruction composed of Z-stack images. We observed significant structural differences in the airways of wild-type and *Svep1^-/-^* lungs, confirming the disorder of the developing airway tree as well as the aberrant duplication of the distal airway tips in *Svep1^-/-^* embryos (Figure 3a).

To quantify the differences in branching morphology in the lungs of mutant vs wild-type embryos, we generated a branching lineage diagram for the RAc lobe at E14.5. Each branch was characterized as one of three branching modes: domain, planar, or orthogonal (Metzger et al., 2008). Branches and direction of growth were mapped to create a lineage diagram in which trifurcations were also indicated (Figure 3b). Orthogonal branching was significantly increased in mutants, contributing 63% of all branches compared to 49% (p<0.01, t-test) in the lungs of *Svep1^+/+^* embryos (Figure 3c). In contrast, planar branching in the RAc lobes was significantly decreased in mutant (5%) compared to wild-type (13%) embryos (p<0.05, t-test; Figure 3c). Trifurcations were more prevalent in *Svep1^-/-^* lungs (10% of all branches) compared to the lungs of *Svep1^+/+^* embryos (4% of all branches) (p<0.05, t-test; Figure 3c and e). An average of 17*%* of first-level domain branches originating from the primary bronchus were ectopic in *Svep1*^-/-^ lungs, while no ectopic branching was observed in the lungs of wild-type embryos (Figure 3d and f). In RAc lobes of wild-type lungs, daughter branches of the primary bronchus sprouted from one of only three directions: anterior, ventral-posterior, and dorsal-posterior. In all the *Svep1^-/-^* lungs analyzed, daughter branches sprouted from the primary bronchus in random directions (Figure 3f). In summary, loss of *Svep1* disrupts airway patterning through decreased planar bifurcations, increased trifurcations, and ectopic domain branching that arise in random directions.

To determine if the ectopic and trifurcated branches in *Svep1^-/-^* lungs were the result of loss of *Svep1*, we analyzed time-lapse images of E12.5 lung explants for 48 hr (Figure 3g, Videos 1–3). We observed ectopic branching arising from the main and secondary branches and trifurcations at the lung tips. Taken together, these results suggest that *Svep1* controls the position and orientation of new airway epithelial buds, defines distal bifurcations regulating the form and structure of the growing epithelium, and is, therefore, critical for determining the bronchial tree architecture and, ultimately, the final shape of the lobes of the lung.

**Video 1.** Embryonic day 12.5 (E12.5) *Svep1^+/+^*^+^ lung explant culture time-lapse images over 48 hr showing normal branching morphogenesis.

**Video 2.** Time-lapse images of embryonic day 12.5 (E12.5) *Svep1^-/-^* lung explant culture over 48 hr demonstrating aberrant branching at the tips and edges of the lung, primarily in the form of trifurcations.

**Video 3.** Time-lapse images of embryonic day 12.5 (E12.5) *Svep1^-/-^* lung explant culture over 48 hr show ectopic branching on the left lobe.

### SVEP1 recombinant protein inhibits airway branching in normal E12.5 lung explants and restores normal branching in Svep1^-/-^ lungs

**Figure 4.**
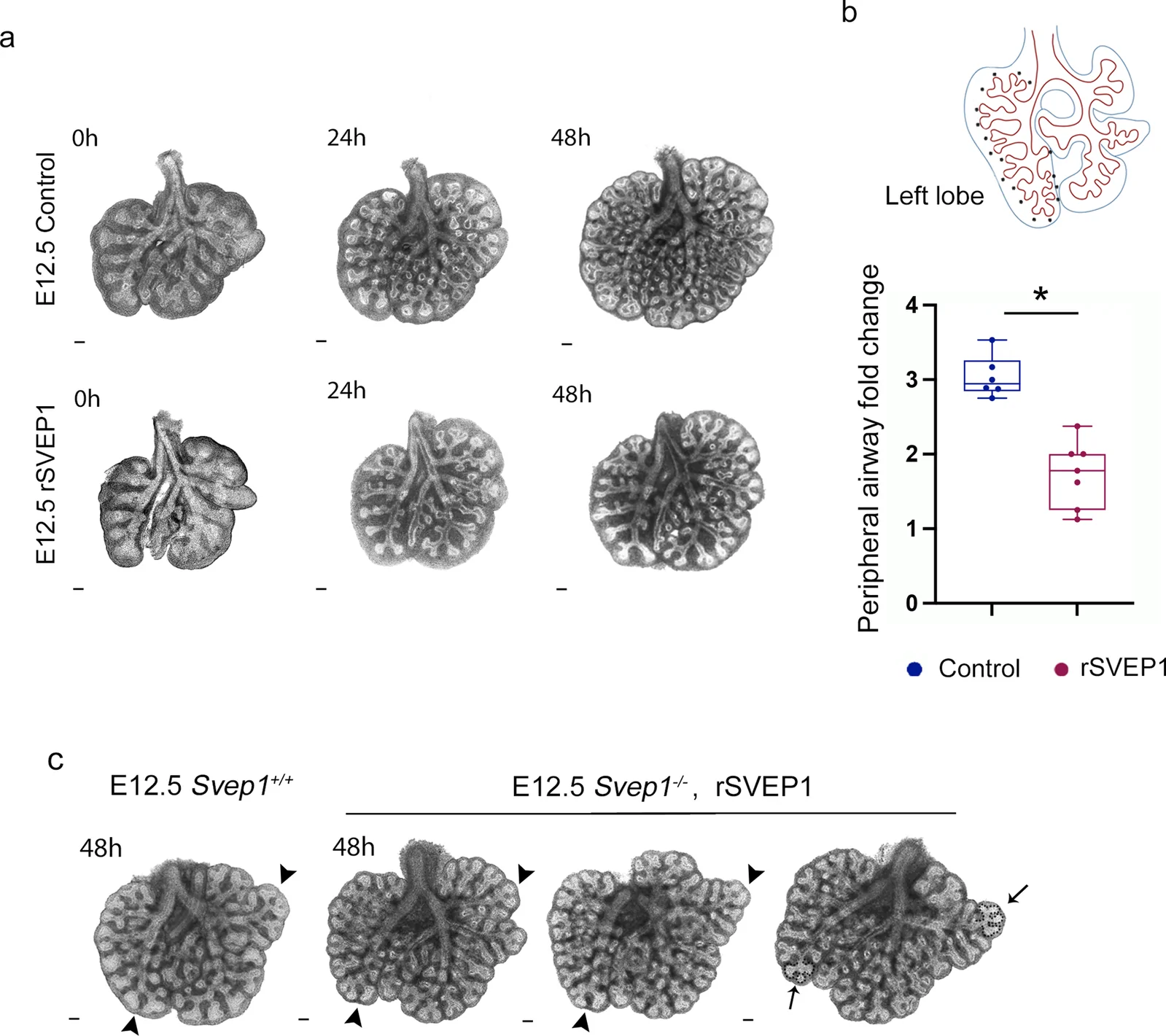
SVEP1 treatment modulates airway branching in embryonic day 12.5 (E12.5) lung explants. (**a**) Images of lung explants from E12.5 *Svep1^+/+^* embryos cultured for 48 hr. Images of lung explants from E12.5 *Svep1^+/+^* embryos cultured for 48 hr show a significant reduction in branching (control, top row) when treated with SVEP1 peptide (bottom row). (**b**) Plot showing peripheral airway counts of a left lung lobe after 48 hr cultured with and without SVEP1 treatment. This panel was created using BioRender.com. (**c**) *Svep1^-/-^* lung explants treated with SVEP1 for 48 hr show a reduction in trifurcations compared to untreated lung explants. Black arrowheads indicate bifurcation; arrows indicate trifurcations (mean ± SEM; two-tailed unpaired t-test; control n=6, rSVEP1, n=7; p<0.05). Scale bars: 100 µm (**a, c**). Figure 4—source data 1.Peripheral airway fold change data for a left lung lobe explant following 48 hr cultured with and without SVEP1 treatment (Figure 4b, panel b).

The increased branching observed in the distal airways of *Svep1^-/-^* embryos suggests that SVEP1 normally acts as an inhibitor of lung branching morphogenesis and is essential for regulating lung airway budding. To further investigate the function of *Svep1* in branching morphogenesis, we treated lung explants of wild-type E11.5–12.5 embryos for 48 hr with a recombinant SVEP1 (rSVEP1) protein. Time-lapse images revealed significantly decreased branching in the treated lung explants (Figure 4a; Videos 4 and 5), quantified at the peripheral airway tips (Figure 4b). SVEP1 branching inhibition was more profound at earlier stages (11.5) (data not shown). When lung explants from E12.5 *Svep1^-/-^* embryos were treated with rSVEP, distal ectopic and trifurcated branches were reduced, and observed in only one of six explants (Figure 4c). The *other Svep1^-/-^* explants exhibited distal bifurcation that resembled wild-type lungs (Figure 4c).

**Video 4.** Control embryonic day 12.5 (E12.5) *Svep1^+/+^*^+^ time-lapse images for the rSVEP1-treated lungs (Video 5), showing normal branching.

**Video 5.** Time-lapse images of embryonic day 12.5 (E12.5) *Svep1^+/+^*^+^ lung explants treated with rSVEP1 protein for 48 hr, demonstrating decreased secondary branching.

### The loss of Svep1 leads to defects in smooth muscle and bronchial airway development

**Figure 5.**
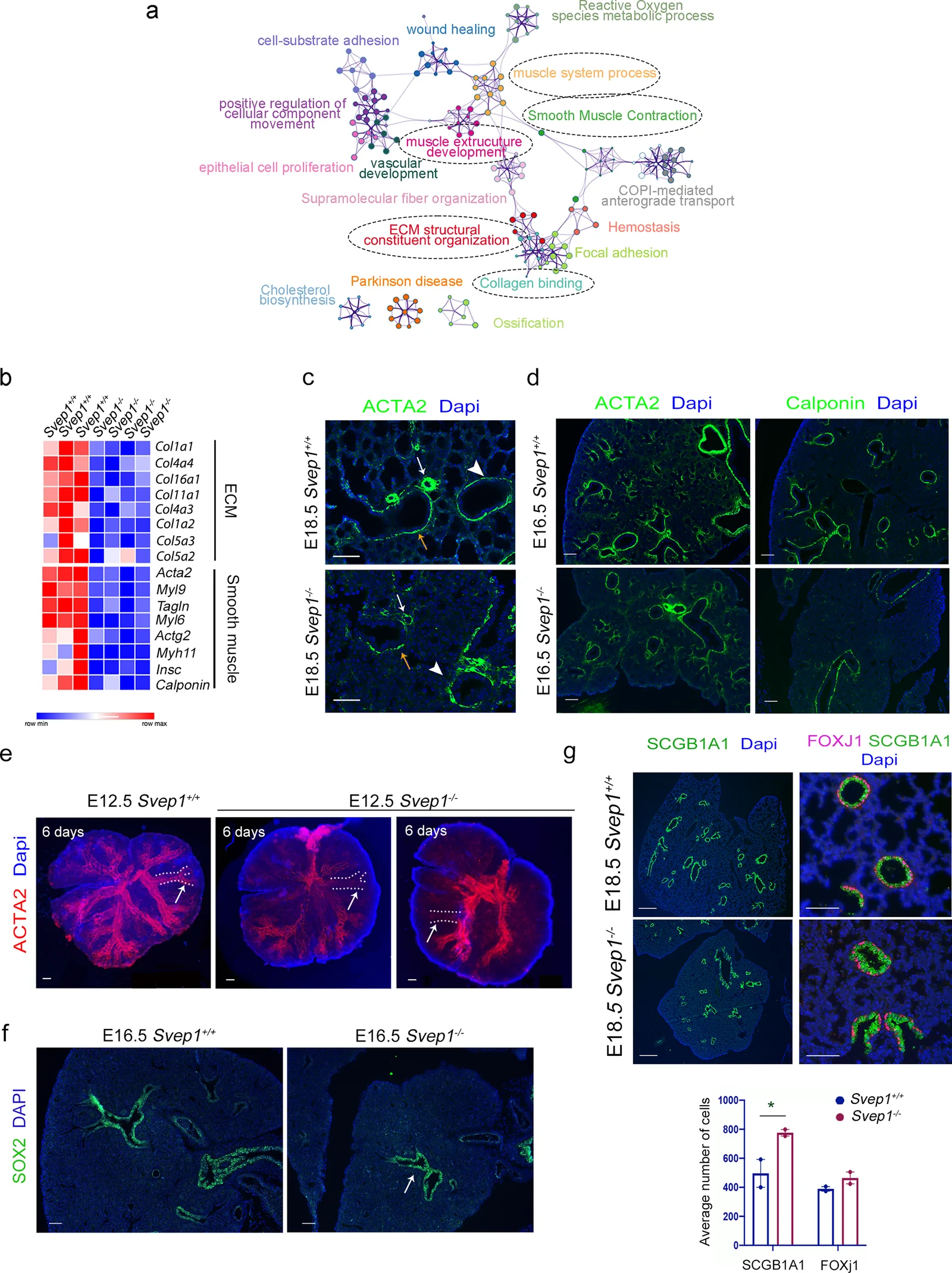
Impaired smooth muscle and airway epithelium differentiation in *Svep1^-/-^* embryonic lungs. (**a**) Interactome of downregulated genes in lungs of embryonic day 18.5 (E18.5) *Svep1^-/-^* embryos with clusters labeled by enriched annotation terms. (**b**) Gene expression heatmap from RNA sequencing shows that significantly downregulated genes in lungs from *Svep1^-/-^* embryos are associated with collagen and smooth muscle genes. (**c**) ACTA2 and calponin staining at E16.5. (**d**) ACTA2 and calponin localization in lung at E18.5 (white arrowheads indicate distal respiratory bronchioles; orange arrows indicate proximal bronchioles; white arrows indicate blood vessels). (**e**) ACTA2 staining (red) in wild-type and *Svep1^-/-^* E12.5 lung explants, each cultured for 6 days, showed a reduction in smooth muscle cell formation in secondary and tertiary bronchi of the mutants (white arrows). (**f**) SOX2 immunofluorescence staining marking the progenitor cells of the epithelium in the proximal airways. Arrow marks the proximal airway at the core of the *Svep1^-/-^* lung. (**g**) Immunofluorescence of the club cell marker SCGB1A1 (green) highlights the small proximal airways in mutant lungs. SCGB1A1 (green) co-localization with the ciliated cell marker FOXJ1 (pink) reveals a multicellular airway epithelium composed primarily of club cells. In *Svep1^-/-^* embryos, the bronchiolar lumina are narrower than *Svep1^+/+^* (highlighted in white). Quantification shows an increased average of number of club cells (SCGB1A1) but not of ciliated cells (FOXJ1) (average counts ± SEM; t-test, p<0.05, n≤3). Scale bars: 200 µm or 100 µm. Figure 5—source data 1.Counts of SCGB1A1 and FOXJ1 positive cells in lungs of Svep1wild type and mutant embryos (Figure 5g).

To characterize further the lungs of *Svep1^-/-^* embryos at the saccular stage, we conducted RNA-seq of whole lungs of *Svep1^+/+^* and *Svep1^-/-^* embryos. Term enrichment analysis of the functional annotations for the differentially expressed genes revealed that genes associated with cell mobility and adhesion, epithelial proliferation, ECM organization, and collagen binding were significantly downregulated in *Svep1^-/-^* lungs (Figure 5a and b).

**Figure 5—figure supplement 1.**
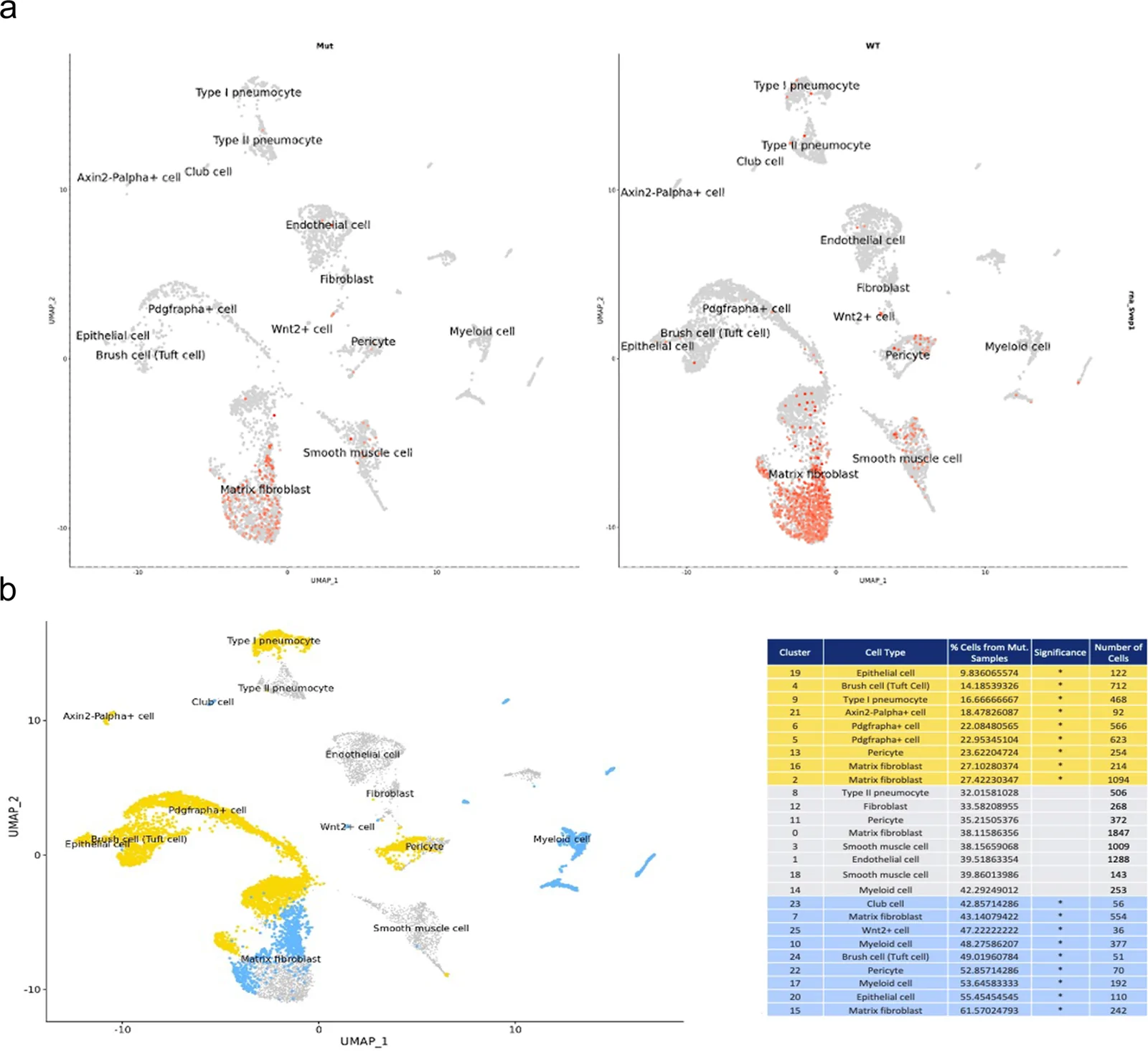
UMAP clustering of single-cell RNA sequencing (scRNA-seq) generated from lungs of Svep1^+/+^ and Svep1^-/-^ embryos at embryonic day 18.5 (E18.5). (**a**) UMAP clustering showing matrix fibroblast cells have the highest expression of Svep1 (reddish orange). (**b**) Clusters deficient in mutant cells compared to cells from wild-type samples are shaded in yellow. Clusters with enrichment of cells from mutant lung samples are shaded in blue.

The RNA-seq data from the whole RCa lobe at E18.5 revealed that genes associated with muscle development were significantly downregulated in *Svep1^-/-^* lungs, including *Acta2* (actin alpha 2), which is necessary for smooth muscle differentiation, and *Cnn1* (calponin1), a marker for mature smooth muscle (Figure 5b). Lungs labeled with ACTA2 and particularly CNN1 confirmed the reduction in distal smooth muscle differentiation in the mutant at E18.5 (Figure 5c) evident around the proximal airways and adjacent blood vessels, particularly CNN1 expression (Figure 5c). The expression of ACTA2 was noticeably reduced also in lobular bronchioles in ex vivo organ culture of E12.5 *Svep1^-/-^* lung explants that were allowed to grow in vitro for 6 days (Figure 5e), confirming that *Svep1* is necessary for proper peribronchial smooth muscle differentiation, which is defective in the lungs of *Svep1* mutant embryos before saccular development.

To investigate lung airway development in *Svep1^-/-^* mutants further, we examined cellular differentiation of the proximal epithelium by staining with the proximal epithelium progenitor SRY-box 2 (SOX2) marker at the end of branching morphogenesis (E16.5) (Figure 5f) and with both club cell secretoglobin 1a1 (SCGB1A1) and ciliated cell forkhead box protein J1 (FOXJ1) markers at E18.5 in the proximal airway epithelium (Figure 5g). In mutants, SOX2 protein was predominantly localized in the lung core (Figure 5f), suggesting that the airway epithelium follows a normal developmental pattern from proximal to distal at E16.5, with most proximal epithelium undergoing typical development. SCGB1A1 staining revealed that *Svep1^-/-^* deficient embryos displayed smaller distal bronchioles, accompanied by disorganized and densely packed SCGBA1+ epithelial cells, leading to an increase in SCGBA1+ cells (Figure 5g). In contrast, the number of FOXJ1+ cells in mutants did not appear to differ from those in wild-type lungs (Figure 5g). Although *Scgba1* mRNA expression did not significantly differ between mutant and wild-type animals, likely due to normal epithelial development in the most proximal airways, the scRNA-seq data indicated that the club cell cluster was enriched in *Svep1^-/-^* lungs (Figure 5—figure supplement 1b). These findings highlight that normal airway structure relies on functional *Svep1*, as the absence of *Svep1* leads to anomalies in bronchial epithelium morphology and an increased number of club cells.

### Altered FGF signaling is a contributing factor to the abnormalities in smooth muscle development observed in Svep1^-/-^ lungs

**Figure 6.**
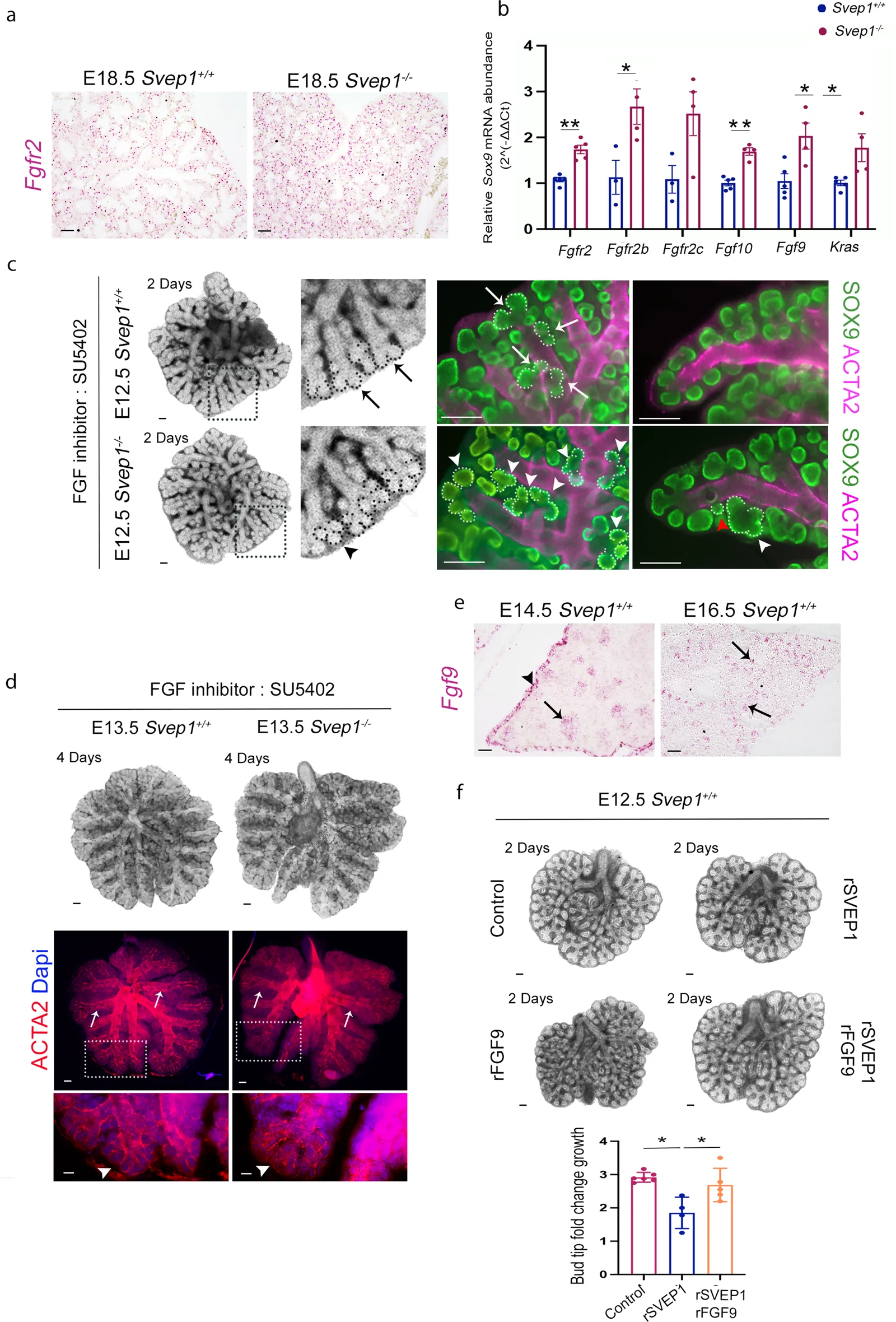
Enhanced FGF signaling in *Svep1^-/-^* embryos contributes to smooth muscle differentiation and branching defects, and FGF9 rescues SVEP1 inhibition of branching. (**a**) In situ hybridization demonstrates increased expression of *Fgfr2* in *Svep1^-/-^* mutants. (**b**) Relative transcript abundance of *Fgfr2* (and its isoforms *Fgfr2b* and *Fgfr2c*), *Fgf10*, *Fgf9,* and *Kras* showing increased expression in *Svep1^-/-^* mutant embryos at embryonic day 18.5 (E18.5). (**c**) *Svep1^+/+^* and *Svep1^-/-^* lung explants from E12.5 embryos treated ex vivo with the FGF signaling inhibitor SU5402 (2.5 µg/ml). Black arrows point to normal tip morphology in *Svep1^+/+^* lungs. The black arrowhead points to distal tip trifurcations that persist in lung explants from *Svep1^-/-^* embryos. SOX9 (green) and ACTA2 (purple) staining confirm the branching anomalies (highlighted in the white dotted areas) in *Svep1^-/-^* lung explants. White arrows mark normal bifurcations, white arrowheads indicate trifurcations and distal budding; the red arrowhead highlights ectopic budding not affected by the induction of FGF. (**d**) E12.5 lung explants treated with SU5402 (2.5 µg/ml) for 4 days demonstrate reduced ACTA2 localization in distal airways of *Svep1^-/-^* lungs compared to wild-type lungs. (**e**) *Fgf9* mRNA expression in wild-type (E14.5) and mutant lung (E16.5). (**f**) Wild-type E12.5 lung explants after 48 hr: untreated control (top left); treated with SVEP1 alone (top right); FGF9 alone (bottom left); combination of SVEP1 and FGF9 (bottom right). The combination restores a normal bud tip phenotype (average counts ± SEM; t-test, p<0.01 and <0.05, n>3). Scale bars: 100 µm (**a**, **c**, **d**, **e**). Figure 6—source data 1.Transcript abundance of Fgfr2, Fgfr2 isoforms, and Kras in Svep1-/- mutant embryos at E18.5 (Figure 6b). Figure 6—source data 2.Lung bud tip growth fold change in treated and untreated Svep1 wild type E12.5 lung explants (Figure 6f).

RNA-seq data from whole lungs revealed that *Fgfr2* expression was significantly increased in *Svep1^-/-^* embryos at E18.5. *Fgfr2* is expressed in the epithelium of the normal lung with decreased expression after E17.5 (Beauchemin et al., 2016). In *Svep1^-/-^* embryos, however, ISH showed that levels of expression of *Fgfr2* transcripts persisted in the lungs of mutants, even at E18.5, compared to levels in the lung of *Svep1^+/+^* embryos (Figure 6a). Results from RT-qPCR showed a significant increase in RNA expression levels of *Fgfr2* and other members of the FGF signaling pathway, including *Fgf10*, *Fgf9*, and *Kras,* in the lungs of *Svep1* mutants at E18.5 (Figure 6b) but not at E14.5 or E16.5 (data not shown). *Fgfr2* has two isoforms, *Fgfr2b* and *Fgfr2c*. Only *Fgfr2b* was significant at E18.5 (Figure 6b). Furthermore, *Svep1^-/-^* mutants showed no change in the expression levels of important genes involved in lung development, such as *Tgfb1*, *Bmp*4, and *Shh at* E14.5, E16.5, and E18.5 (data not shown).

FGF10/FGFR2b signaling is necessary for airway outgrowth and promotes epithelial progenitor proliferation and migration (El Agha and Bellusci, 2014; Yin and Ornitz, 2020). FGF10+ cells are progenitors to parabronchial smooth muscle cells, and this progenitor status is maintained by mesothelial-derived FGF9, which inhibits smooth muscle differentiation. Smooth muscle progenitors become gradually displaced from distal to a proximal position alongside the bronchial tree in the mouse lung (del Moral et al., 2006; Yi et al., 2009) during development. To investigate if FGF signaling is involved in *Svep1^-/-^* branching defects and airway smooth muscle differentiation, we treated lung explants with the FGF pathway inhibitor SU5402 at a low enough dose to allow for some branching activity. SU5402 is a fibroblast growth factor receptor 1 (FGFR1) tyrosine kinase inhibitor that blocks the FGF signaling necessary for smooth muscle differentiation, leading to more smooth muscle around the airway (Goodwin et al., 2019). As expected, we observed branching defects in the control and *Svep1^-/-^* lung explants after 48 hr of treatment (Figure 6c). Immunofluorescence SOX9 and ACTA2 revealed normal distal airway bifurcation in explants of *Svep1* wild-type lungs and trifurcations in lung explants from mutant embryos along with ectopic branching and budding anomalies (Figure 6c). After 4 days of FGF signaling inhibition, an increase in smooth muscle coverage around the distal airways, wrapping around the distal tips, was observed in both normal lungs and, to a lesser degree, *Svep1^-/-^* lungs (Figure 6d). Overall, these results suggest that the branching pattern defects in *Svep1* mutants are independent of the canonical FGF pathway, but that smooth muscle differentiation defects are partially mediated by increased FGF signaling.

### SVEP1 and FGF9 interact during branching to direct normal lung development

*Fgf9* is essential for mouse lung development, and both loss- and gain-of-function alleles of *Fgf9* result in branching defects (Colvin et al., 1999; del Moral et al., 2006; Yin et al., 2011; Lin et al., 2006). *Fgf9* is expressed in both the lung mesothelium and distal epithelium during early-to-mid branching morphogenesis. *Fgf9* levels in the mesothelium are high at E12.5, but at E14.5 *Fgf9* is limited to the distal epithelium (Colvin et al., 1999; del Moral et al., 2006). We found that *Fgf9* is normally expressed in mesothelium and epithelium at E14.5 and exclusively in the distal airway tip epithelium of E16.5 lungs (mid-to-late branching) (Figure 6e).

Overexpression of *Fgf9* causes branching defects that are characterized by dilatation of the distal airways (del Moral et al., 2006; Yin et al., 2011; Yin and Ornitz, 2020; White et al., 2006), a phenotype that is opposite to the multi-budding phenotype of *Svep1^-/-^* lungs at the pseudoglandular stage (Figure 2c). Furthermore, overexpression of *Svep1* (Figure 4a and b) and loss of *Fgf9* function (Colvin et al., 1999; del Moral et al., 2006) inhibit branching. To investigate whether SVEP1 and FGF9 interact during branching morphogenesis, we treated normal lung explants with exogenous SVEP1 and FGF9 alone and in combination for 2 days. SVEP1-cultured explants showed a significant decrease in branching (Figure 6f). FGF9-cultured explants exhibited dilated distal airways (Figure 6f). Lung explants treated with both SVEP1 and FGF9 resulted in normal branching and distal airways (Figure 6f). That the treatment with combined SVEP1 and FGF9 restored branching and distal airway morphologies suggests that an interaction between SVEP1 and FGF9 is necessary for normal airway development.

### Svep1 is necessary for cellular differentiation and saccule maturation

**Figure 7.**
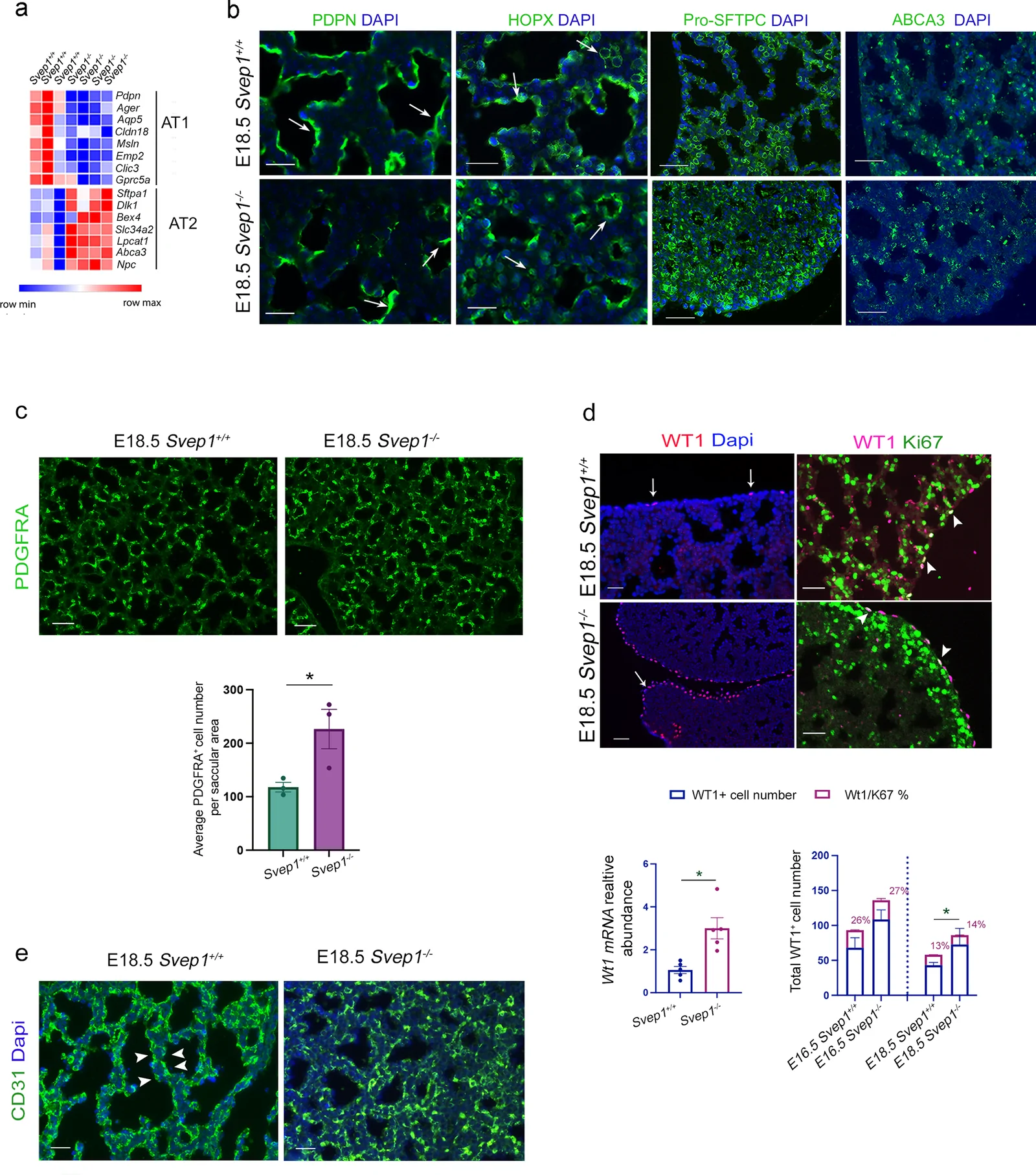
Saccular cellular and structural defects in *Svep1^-/-^* embryos at embryonic day 18.5 (E18.5). (**a**) Expression heatmaps of selected genes associated with alveolar AT1 and AT2 cells show that *Svep1^-/-^* lungs have lower expression of AT1 genes compared to *Svep1^+/+^* lungs and higher expression of AT2-associated genes. (**b**) Immunofluorescence of PDPN, HOPX, Pro-SFPC, and ABCA3 in the lungs of E18.5 *Svep1*^+/+^ and *Svep1*^-/-^ embryos. Arrows indicate airway bud stalks. (**c**) PDGRFA+ (green) staining at E18.5. Plots of the average number of PDGFRA+ cells (average counts ± SEM; t-test, p<0.05, n=3). (**d**) Immunofluorescence of the mesothelial progenitor marker WT1 (red) with KI67 (green) staining indicating actively dividing WT1+ progenitors (white arrows). Plot of the number of WT1+ and WT+/KI67+ cells at E16.5 and E18.5. *Svep1^-/-^* lungs have more WT1+ cells than *Svep1^+/+^* lungs at E18.5; however, the number of WT1+ KI67 lungs is unchanged between *Svep1^+/+^* and *Svep1^-/-^* lungs. (**e**) Labeling of endothelial cells with CD31 illustrating the double layer of the saccular microvasculature in normal lungs (arrowhead) and disorganized microvasculature in *Svep1^-/-^* lungs at E18.5. Scale bars: 250 µm (**b**); 100 µm (**c**, **d**); 5 µm (**e**, **h**). Scale bars: 25 µm (**c**, **e**); 50 µm (**b**); 100 µm *(***c**). Figure 7—source data 1.Average counts of PDGFRA+ cells in Svep1 wild type ad mutant lungs at E18.5 (Figure 7c, panel c). Figure 7—source data 2.Relative transcript abundance of WT1 in lungs of Svep1 wild type and mutant embryos at E18.5 (Figure 7d). Figure 7—source data 3.Counts of WT1+ cells in lungs of Svep1 wild type and mutant embryos at E18.5 (Figure 7, panel d).

In *Svep1* mutants, alveolar saccules are either absent or underdeveloped, indicating that there is a disruption in the normal alveolar differentiation process. Our transcriptomics data indicate an increase in expression of AT2-related genes and a decrease in expression of AT1-related genes in *Svep1^-/-^* lungs at E18.5 (Figure 7a). Immunolabeling with AT1 markers (PDPL and HOPX) and AT2 markers (SFTPC and ABCA3) reveals reduced PDPL and HOPX1 positive domains, expanded SFTPC expression, and intensified expression of ABCA3 in the central saccules at E18.5 (Figure 7b). Additionally, SFTPC expression correlates with SOX9, indicating an immature AT2 cell fate phenotype in mutants (Figure 7—figure supplement 1a). These findings underscore that *Svep1* deficiency disrupts the balance of alveolar epithelial differentiation, favoring an AT2 cell fate. This effect is particularly pronounced in the lung periphery, which retains an AT2 progenitor phenotype reminiscent of the pseudoglandular stage.

**Figure 7—figure supplement 1.**
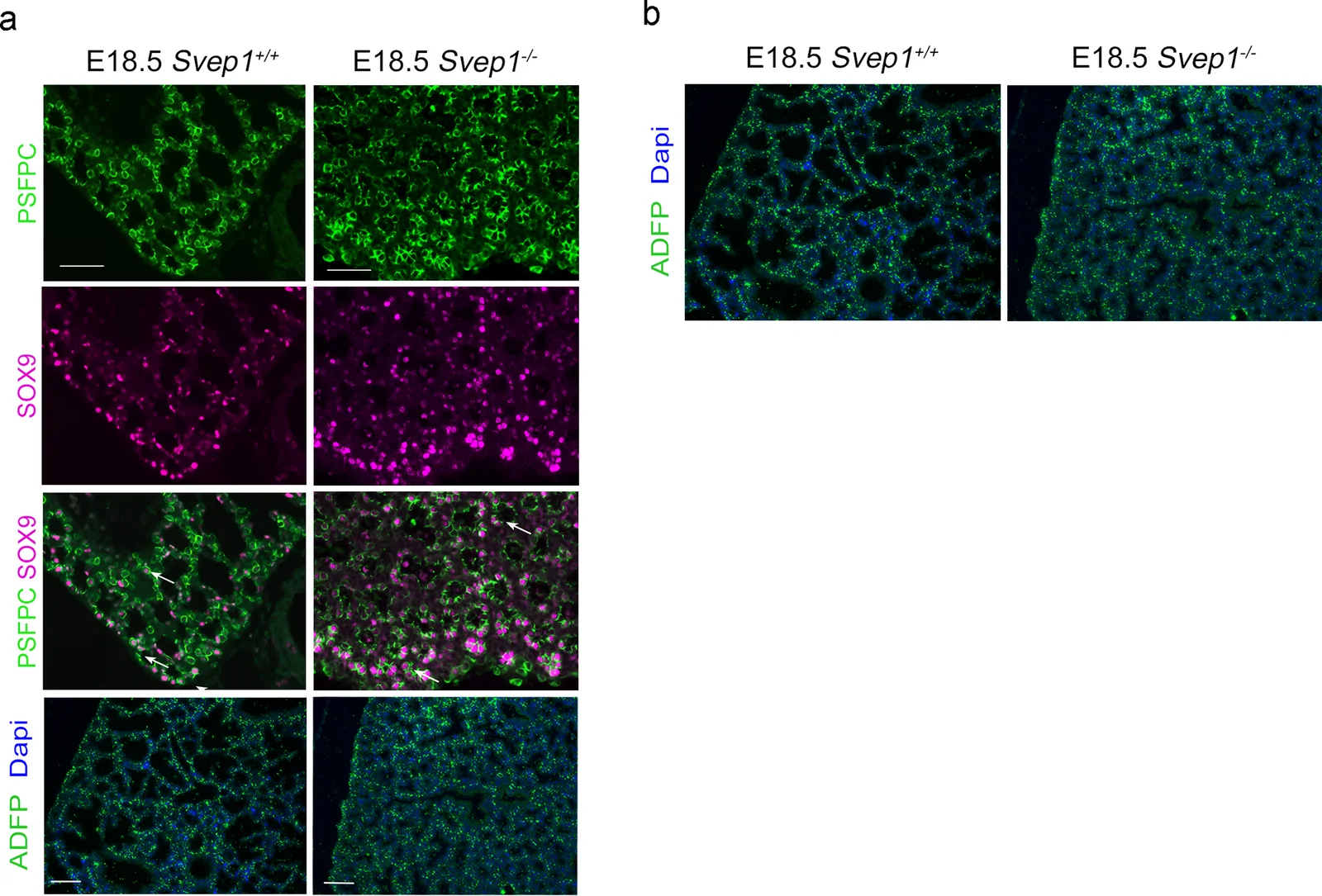
SOX9, SFTPC, and PLIN2 (ADFP) immunohistochemistry in lungs of embryonic day 18.5 (E18.5) embryos. (**a**) Epithelial localization of surfactant-associated protein C (SFTPC; green) and SRY-box 9 (SOX9; purple) expression in the distal airway tips at E18.5. Arrows indicate the co-localization of SFPC and SOX9. (**b**) Perilipin 2 (PLIN2, aka ADFP) marks lipofibroblasts in the lung mesenchyme. Expression of the Plin2 (Adfp) gene in the lungs of Svep1 mutant and wild-type E18.5 embryos was not statistically different. Scale bars: 100 µm.

**Figure 7—figure supplement 2.**
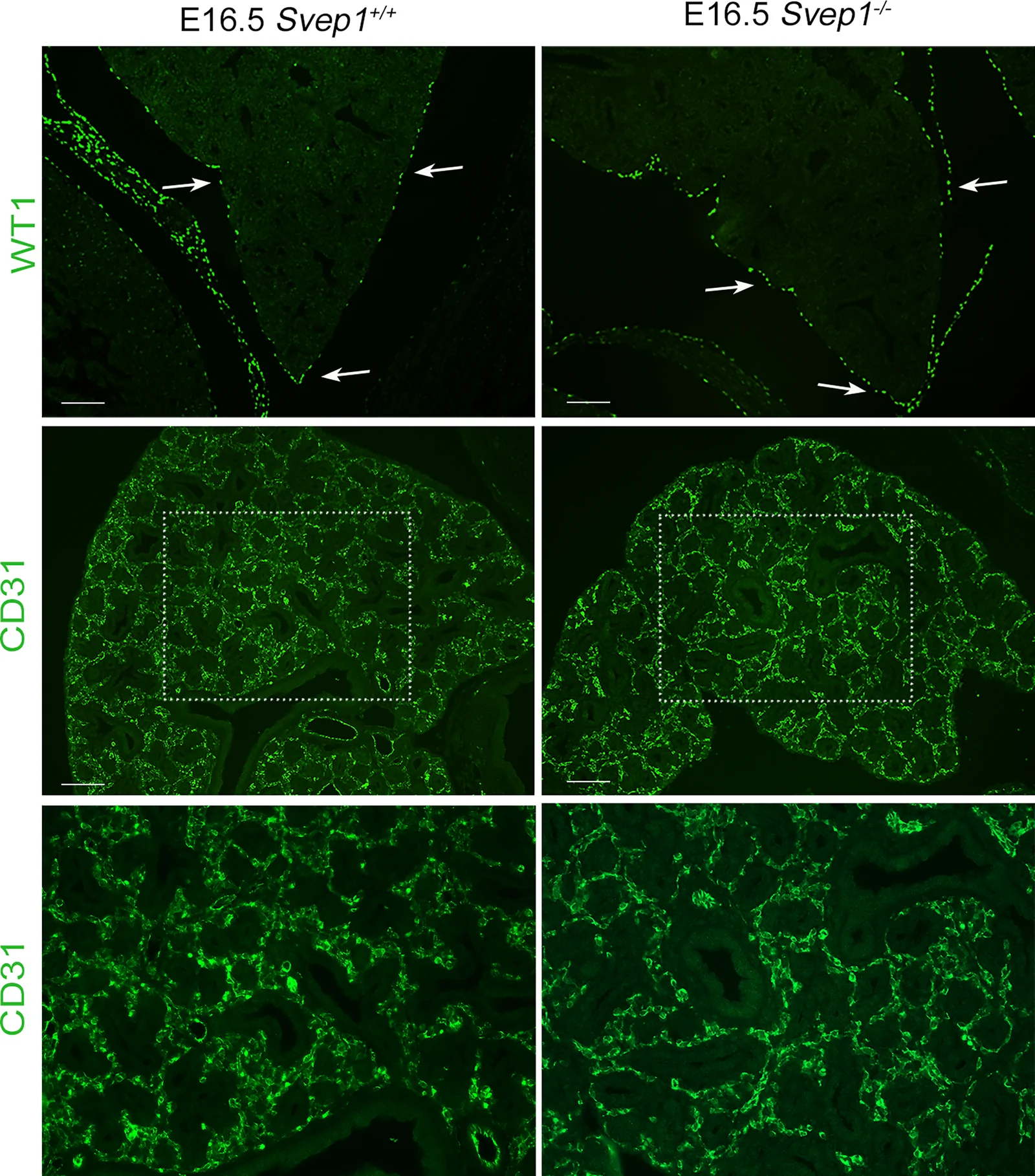
WT1 and CD31 expression in embryonic day 16.5 (E16.5) lungs of Svep1^+/+^ and Svep1^-/-^ embryos. (**a**) Mesothelial localization of WT1 (green) at E16.5, with arrows indicating WT1+ cells around the lung. (**b**) CD31 (green) localization marking the developing microvasculature in lungs at E16.5. Bottom panels for CD31 labeling are from regions outlined in white dotted areas in the panel above. Scale bars: 100 µm.

Alveolar ACTA2 positive myofibroblasts transiently differentiate from their PDGFRA positive progenitors during septation which, during normal murine lung development, are located at the base of saccules in the regions of future alveoli, where they invaginate toward the saccule lumen (McGowan et al., 2008; Branchfield et al., 2016). To assess if *Svep1^-/-^* lungs exhibit defects in alveolar myofibroblast progenitor formation, we stained E18.5 tissues with PDGFRA, revealing a significant increase in the number of PDGFRA^+^ cells per saccular tissue in mutants (Figure 7c). While RNA-seq analysis showed no significant difference in *Pdgfra* gene expression between mutant and wild-type lungs, the scRNA-seq data revealed a deficiency in *Pdgfra^+^* cells in the lungs of *Svep1^-/^*^-^ embryos (Figure 5—figure supplement 1). The expression of *Pdgfa*, the PDGRA ligand crucial for septation, myofibroblast differentiation, proliferation, and migration (Gouveia et al., 2018; Betsholtz, 2004), was reduced in *Svep1^-/-^* embryos at E18.5 in the bulk RNA-seq data (Log_2_(FC) = –0.19318, adjusted p-value<0.05, t-test). PDGFRA+ cells, which are known to give rise to alveolar lipofibroblasts characterized by lipid-filled vesicles, exhibited no differences in *Plin2* (*perilipin 2*, also known as adipophilin; *Adfp*) expression between mutant and wild-type lungs at E18.5 (Figure 7—figure supplement 1). These findings show that *Svep1* expression is essential for regulating alveolar myofibroblast progenitor formation. Notably, despite the absence of primary septation, *Svep1^-/-^* mutants exhibit a broad myofibroblast progenitor fate (PDGFRA+ cells). This increase may arise from an augmented number of distal airways and/or defects in the myofibroblast differentiation process itself.

The lungs are covered by a monolayer of flat mesothelial cells that differentiate from round Wilms’ tumor transcription factor 1 (WT1) positive cells that surround the lung buds during early branching (Dixit et al., 2013). *Wt1* expression normally decreases during development (Dixit et al., 2013), whereas the hypercellular mesothelium of the *Svep1^-/-^* lungs was enriched in WT1+ mesothelial progenitors and had increased *Wt1* gene expression (Figure 7d). To determine if the expansion of mesothelial cells was due to an increase in cellular proliferation, we co-labeled with WT1 and KI67 (Figure 7d). Although there was a significant increase in the number of WT1+ cells in *Svep1^-/-^* lungs (Figure 7d, left), the percentage of WT1+ that were also KI67+ was not significantly different between normal and mutants at E16.5 (Figure 7d, right), indicating that the disruption of mesothelial differentiation in mutants is not due to an increase in proliferation.

RNA-seq data from the whole lung revealed that vascular development genes are downregulated in the lungs of *Svep1^-/-^* embryos compared to wild-type controls (Figure 6a). To determine, therefore, if lung microvasculature formation is affected in *Svep1^-/-^* embryos, we stained E16.5 (Figure 7—figure supplement 2b) and E18.5 (Figure 7e) lungs with PECAM1 (CD31). At E16.5, lung microvasculature looks normal (Figure 7—figure supplement 2b); however, at E18.5, the microvasculature was disorganized and failed to form the double-layer pattern characteristic of the saccular stage (Figure 7e). These results indicate that *Svep1* is necessary for lung microvasculature plexus formation, which is consistent with previous findings that *Svep1* plays a role in lymphatic vascular development and remodeling in mice and explains why disruption of *Svep1* results in severe edema in embryos (Karpanen et al., 2017; Morooka et al., 2017).

## Discussion

**Figure 8.**
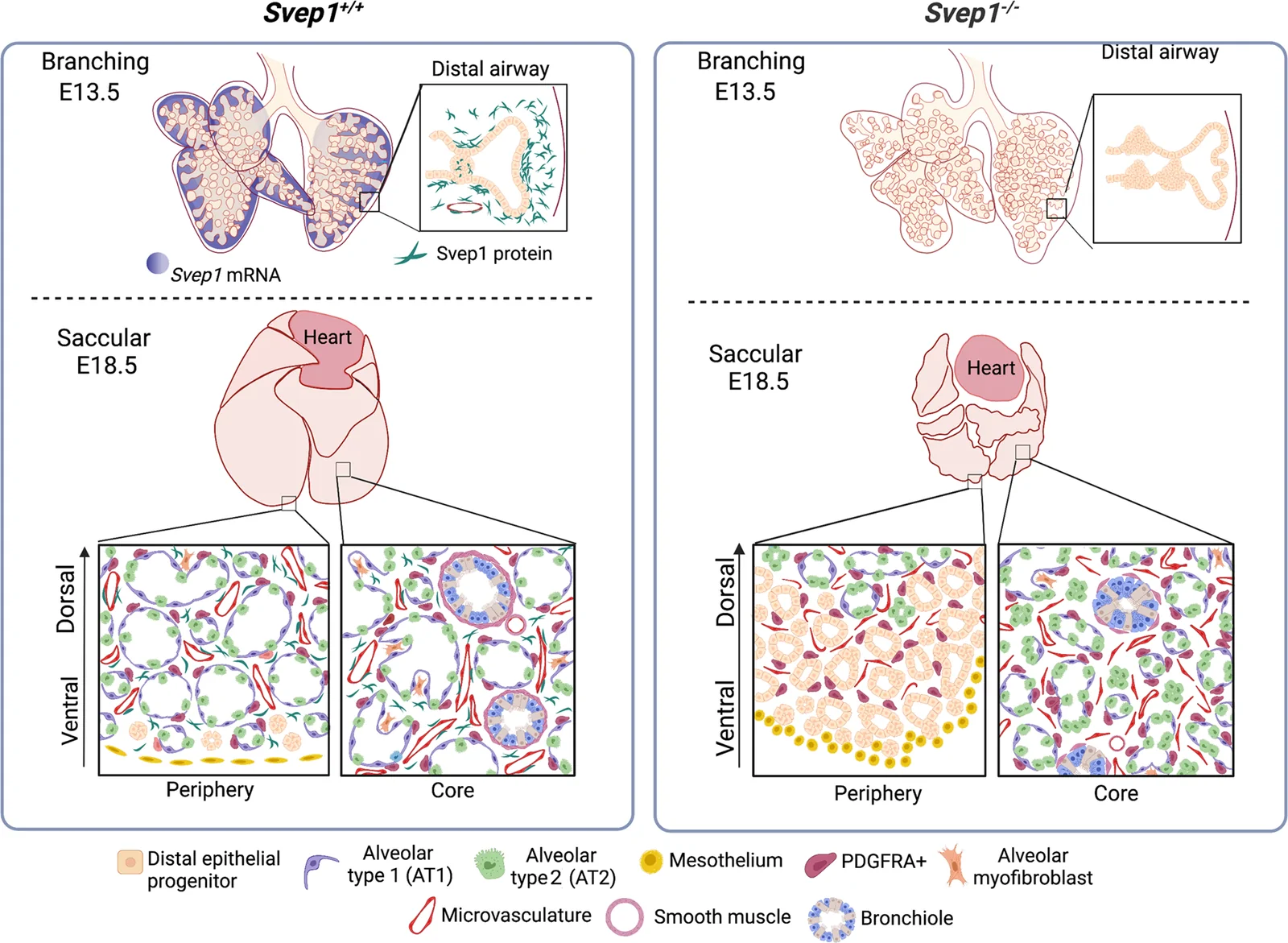
Graphical summary of normal and *Svep1^-/^*^-^ embryonic lung phenotypes during branching morphogenesis and saccular formation. Created using BioRender.com.

While previous studies have led to important insights into the genetic and molecular processes that underlie lung development, our understanding of the factors that regulate the bronchial tree’s stereotypic structure and the subsequent transition from branching to saccular formation remains incomplete. The study reported here revealed, for the first time, that *Svep1* function is necessary to preserve the stereotypic pattern of the bronchial tree, contributes to the transition required to end branching morphogenesis, and promotes alveolar differentiation and maturation (Figure 8). The results also suggest that *Svep1* regulates the FGF signaling pathway for smooth muscle differentiation and that the interaction between SVEP1 and FGF9 is essential for normal airway development. Although further investigation is needed to uncover molecular mechanisms, the findings reported here provide new insights into the role the ECM plays in governing lung morphogenesis.

Our work shows that *Svep1* plays a significant role in regulating the branching program in the developing lung. The loss of *Svep1* expression leads to disordered airway tips, particularly in the distal regions where *Svep1* expression is normally high. Our results demonstrate that SVEP1 acts as a negative regulator of lung branching, inhibiting the growth of new branches in lung explants. The effects of SVEP1 on the lung bud epithelium suggest a role in shaping the lobes by restricting the potential for aberrant branching. We hypothesize that early in lung development, the local genetic program driven by FGF from its signaling centers is sufficient to direct local branching; however, as lung airway complexity increases, this local signaling becomes diffuse and may activate the entire susceptible SOX9^+^-directed epithelial branching. Therefore, negative regulators, like SVEP1, act to restrict excessive branching while ensuring proper airway patterning. Notably, at the pseudoglandular stage, lungs of *Svep1^-/-^* embryos show no significant differences in the expression of essential branching genes. This suggests that *Svep1* may not be the primary regulator of the overall genetic branching program but rather a modulator of the timing and extent of branching events. *Svep1* most likely directs branching through localized signaling without significantly impacting genes fundamental for branching morphogenesis (e.g. *Fgf10*, *Shh*, and *Bmp4*), allowing early lung developmental processes while maintaining later appropriate branching morphology.

The recovery of SVEP1 branching patterns in lung explant cultures following exogenous treatment of FGF9 suggests a potential physical interaction between SVEP1 and FGF9 in the regulation of branching similar to the SVEP1-ANG2 during the remodeling of lymphatic vessels (Morooka et al., 2017; Sato-Nishiuchi et al., 2023). It is plausible that SVEP1 interacts with FGF9 signaling locally in a particular distal epithelial progenitor to modify the overall branching program.

The disordered airways in the lungs of *Svep1^-/-^* embryos disrupt the shape of the lung lobes. The disrupted branching program manifests as ectopic budding and trifurcations of distal airways. The ordinarily thin and flat margins of the lung are rounded, and the tips of the lungs are foreshortened in *Svep1^-/-^* embryos. Our 3D model of the RAc lobe showed that branching in *Svep1^-/-^* embryos results in a pattern of dense rosettes generated at multiple branching sites, resulting in a cauliflower-like appearance of the lungs at E18.5. In the lungs of *Svep1^-/-^* embryos, we observed a dorsal-to-ventral maturation gradient with small immature saccules on the dorsal side and narrow airways on the lobulated ventral side. The dorsal-ventral spatial variation in aberrant lung lobe morphology is consistent with previous work, showing that tissue geometry and biomechanical forces influence the genetic branching program during lung development (Blanc et al., 2012; Lang et al., 2021). Our data indicate that the lobe-specific phenotypes in *Svep1^-/-^* lungs are due to a complex interplay of genetic and physical factors and that the ventral lung needs tighter control than the dorsal lung to develop properly to fill the chest cavity together with the heart and diaphragm (ventral) and the body wall (dorsal). Hence, *Svep1* is especially crucial for preserving the basic structure of lung tips and edges during development.

The lungs of *Svep1^-/-^* embryos exhibit impaired cellular differentiation and saccular maturation. *Svep1^-/-^* pups die shortly after birth, with a lung morphology more typical of the pseudoglandular stage with enrichment of SOX9+ progenitor cells. The alveolar epithelium in the lungs of *Svep1* mutant embryos is biased toward AT2 cells, which correlates with the proliferative SOX9+ cell population which, in turn, is maintained by the persistent expression of genes involved in FGF signaling that impedes differentiation of AT1 cells (Volckaert et al., 2013; Gouveia et al., 2018). Furthermore, *Svep1^-/-^* lungs are deficient in HOPX+ and PDPL+ AT1 cells with reduced lung bud stalks potentially leading to proximal airway hypoplasia. *Svep1^-/-^* lungs have reduced smooth muscle differentiation that can be rescued by the inhibition of FGF signaling. The observed increase in SCGBA1+ club cells and the concomitant reduction in bronchiole lumen diameter point to a profound influence of the loss of *Svep1* on airway morphology, in addition to the smooth muscle development defects around the proximal airways. Furthermore, *Svep1^-/-^* lungs show reduced expression of genes related to smooth muscle, including *Acta2*. This deficiency may contribute to the impact of the loss of *Svep1* on septa and airway hypoplasia, influencing the distalization of the airway.

Other studies have found that mechanical pressures, in conjunction with growth factors, are essential for proper alveolar differentiation during development (Rozario and DeSimone, 2010). *Svep1^-/-^* embryos are characterized by chest cavity edema secondary to lymphatic defects at E18.5, which may cause atypical internal lung pressures that could, in turn, contribute to the observed aberrant cell differentiation. Mesothelial cells and alveolar myofibroblasts at E18.5 in *Svep1^-/-^* are also maintained in a progenitor state of immature saccular development marked by increased WT1 and PDGFRA expression in mutants.

Maintenance of the ECM has been shown previously to be essential for normal organ and epithelial tube morphogenesis (Mecham, 2018; Streuli, 1999) and cellular differentiation processes (Streuli, 1999). Our transcriptome analysis indicated that the absence of *Svep1* disrupts the expression of genes associated with cell adhesion, cell motility, and ECM organization. Our transcriptomics data showed that *Col1a1* and the basement membrane collagen *Col4a4* are significantly reduced in E18.5 *Svep1^-/-^* lungs, disruption of which may contribute to the proliferation and differentiation defects that we observed in the lungs of *Svep1^-/-^* embryos in this study.

The transition from branching to differentiation in lung development is essential for establishing the foundation for alveologenesis and has significant clinical ramifications for neonates born with such respiratory deficiencies. Our findings demonstrate the importance of the ECM in directing airway architecture and the transition from branching programs to alveolar differentiation. Additional research into the specific role that *Svep1* plays in preparing the lung for alveologenesis is required to understand more fully the molecular, cellular, and mechanical mechanisms critical to this transition and its potential for targeted therapeutic interventions to improve the outcomes of patients born with impaired or delayed lung development.

## Methods

### Svep1 mutant mice

*Svep1* mutant mice (B6N(Cg)-*Svep1^tm1b(EUCOMM)Hmgu^*/J; MGI:5509058) were generated by the KOMP2 by inserting the L1L2_Bact_P cassette upstream of exon 8 of *Svep1* (Dickinson et al., 2016). This cassette contains a flippase recombinase target (FRT) site followed by a lacZ sequence and a loxP site. The first loxP site is followed by a neomycin resistance gene, a second FRT site, and a second loxP site. A third loxP site is inserted downstream of exon 8. The construct was introduced into embryonic cells, and embryonic stem cell clone HEPD0747_6_B06 was injected into B6(Cg)-*Tyr*^c-2J^/J blastocysts. Resulting chimeric males were bred to C57BL/6NJ female mice to generate heterozygous tm1a (i.e. knockout first) animals (Skarnes et al., 2011). No homozygous mice were recovered at weaning from heterozygous intercrosses. Heterozygous tm1a mice were then bred to B6N.Cg-Tg (*Sox2*-cre)1Amc/J mice to remove the floxed neomycin sequence and exon 8 of *Svep1*. Resulting offspring were bred to C57BL/6NJ mice to remove the *cre*-expressing transgene. Genotyping was performed as described in the Supplementary Methods.

### Histology

Mouse embryos were euthanized by decapitation at E14.5, E16.5, or E18.5. Tails were collected for genotyping. Whole lungs were excised surgically and fixed in 4% paraformaldehyde (PFA) and then embedded in paraffin. Paraffin tissue blocks were sectioned (5 µm) and stained for hematoxylin and eosin (H&E) using standard procedures.

### In situ hybridization

For whole-mount ISH of *Svep1*, a 725 bp segment of the *Svep1* transcript (NM_153366.4; 746–11179) was PCR-amplified (PCR Master Mix, Promega). ISH was performed using methods published previously (Riddle et al., 1993) and developed using BM purple AP substrate (Roche) per the manufacturer’s instructions. 5-µm-thick sections of paraffin-embedded lung tissues were used for ISH of *Fgf10* (NM_446371, 862–1978 nt), *Fgf9* (NM_013518.4, 27–1198 nt), *Fgfr2* (NM_010207, 2-1677 nt), and *Svep1* (NM_022814.2, 2879–3726 nt) using RNAscope 2.0 Red Detection Kit (Advanced Cell Diagnostics), according to the manufacturer’s instructions.

An *Svep1* riboprobe was synthesized with one pair of exon-exon boundary overlapping primers (5’ TGTGGTCCTCCAAGTCACGTA 3’; 5’ CCAGCAGACAGCAGAGTATGT 3’) designed using NCBI’s Primer-BLAST (Ye et al., 2012). The purified PCR fragment was cloned into the pCRII-TOPO TA vector (TOPO TA Cloning Kit, Dual Promoter, Thermo Fisher) and transformed into One Shot TOP10 Chemically Competent Cells (Thermo Fisher). Ampicillin-resistant transformed colonies were selected. The orientation of the insert after linearization of the vector by SpeI restriction digestion (New England Biolabs, Inc) was determined by Sanger sequencing. Sense and anti-sense digoxigenin-11-UTP-labeled probes (DIG RNA Labeling Mix, Sigma-Aldrich) were synthesized using SP6 and T7 RNA polymerases, respectively.

Immunohistochemistry (IHC) on sectioned lung tissue was performed using standard techniques. Details on the antibodies used for IHC are provided in the Supplementary Methods. Antigen retrieval was achieved by heat treatment in a microwave oven for 20 min at low power in 0.01 M sodium citrate buffer at pH 6. For tissues imaged using fluorescence, Alexa Fluor secondary antibodies were used (Life Technologies). Images were obtained with a Nikon80i or Keyence (BZX800) microscope.

### Quantification of lung distal epithelial tips

Fixed whole lungs were washed with PBS and permeabilized in 0.5% Triton X-100 and 5% BSA in PBS for 1 hr at room temperature. SOX9 primary antibody diluted in 1× PBS was incubated overnight at room temperature. Samples were washed and incubated with an Alexa Fluor (Life Technologies, USA) secondary for 2 hr at room temperature, mounted with DAPI mounting medium, and fixed with 4% PFA in PBS for 2–4 hr.

### Tissue clearing for evaluation of branching patterns

Fixed whole lungs from E13.5 embryos were dehydrated in a methanol series, washed with 20% DMSO in methanol, and then re-hydrated with a methanol series. Lungs were then permeabilized with 0.2% Triton X-100 in PBS. Lung tissue clearing was performed using the Visikol Histo kit (Visikol, Inc, NJ, USA) according to the manufacturer’s instructions. Lungs mounted in silicone-imaging chambers were imaged using a Keyence (BZX800) microscope.

### Whole-mount branching immunofluorescence

Lung explants were fixed with 4% PFA, dehydrated in a PBS/methanol series, bleached with 6% hydrogen peroxide (H1009, Sigma) in methanol overnight at 4° C, then re-hydrated in a methanol series. Samples were blocked in PBS + 0.3% Triton X-100+5% normal goat serum for 2 hr and incubated with primary antibodies in blocking solution overnight at 4°C. Samples were washed with PBS + 0.1% Triton X-100+0.1% Tween-20, followed by the addition of Alexa Fluor antibodies (Life Technologies) diluted in PBS + 0.3% Triton X-100 at 4°C overnight. Samples were mounted with DAPI mounting medium, washed, then fixed with 4% PFA in PBS for 2–4 hr. Whole lungs were imaged using a Keyence microscope. A Z-stack image series (BZ-H4XD) was generated for quantification and analyses of branching patterns of embryonic whole lungs. Time-lapse videos were generated to trace the lineages of airway branches for orthogonal and planar bifurcation, and for quantification of trifurcation and ectopic branching. Statistical significance was evaluated using a two-tailed t-test with significance at p<0.05.

### Embryonic lung explant imaging

Lungs from E12.5 or E13.5 embryos were dissected and transferred to the inserts of Corning transwells (Millipore Sigma) containing 2 ml of DMEM/F12 medium supplemented with 1 U/ml penicillin-streptomycin. Lungs were incubated in the chamber and time-lapse images were captured every 10 min over 48 hr using the Keyence BZX800 software (BZ-H4CXT).

### Morphometric analysis of treated lung explants

Lung explants from E12.5 or E13.5 *Svep1^+/+^* and *Svep1^-/-^* embryos were treated for 2 days with a 146 amino acid recombinant protein antigen of *Svep1* (10 µg/ml, NP_0735725.2, region 874–971, Novus Biologicals), for 2–4 days with and without an FGFR2 kinase inhibitor (2.5 µM SU5401, Pfizer) signaling and for 2 days with FGF9 (200 µM; R&D Systems) or FGF9+SVEP1 recombinant protein.

The fold change of epithelial branches was determined by manual counting of individual branches compared to epithelial buds emerging from the lung epithelium at various intervals throughout a time-lapse experiment using ImageJ (Schneider et al., 2012). Lung buds were categorized as peripheral branches only if they appeared at the most distal end of the secondary bronchi. Quantification was performed for the left lobe only. Statistical significance of the counts was evaluated using a two-tailed t-test with significance at p<0.05.

### RNA-seq and analyses

Total RNA was isolated from whole embryonic lungs at E18.5 using the RNeasy Mini Kit (QIAGEN, Valencia, CA, USA) according to the manufacturer’s instructions. Double-stranded cDNA was synthesized from total mouse lung RNA using the iScript cDNA Synthesis Kit from Bio-Rad following the manufacturer’s instructions. RNA samples were sent to Novogene Co. Ltd for sequencing on the Illumina platform (see Supplementary Methods). Annotation term enrichment for pathways and function for differentially expressed genes was performed using Metascape (http://metascape.org; Zhou et al., 2019), VisuaL Annotation Display (VLAD; Richardson and Bult, 2015; http://proto.informatics.jax.org/prototypes/vlad/), and the Gene Ontology term enrichment tool (http://geneontology.org/docs/go-enrichment-analysis/). Heatmaps of gene expression were generated using the Morpheus matrix visualization and analysis software (https://software.broadinstitute.org/morpheus/).

### scRNA-seq and analysis

Single-cell suspensions for scRNA-seq were prepared from E18.5 embryos, sequenced, and processed as described in the Supplementary Methods. The Seurat software package was used to analyze the processed data (Hao et al., 2021). To determine the cell populations most impacted by the loss of *Svep1*, the distribution of cell populations in each experimental group was determined by calculating the ratio of each Seurat cluster to the total number of cells from an experimental group. A t-test of the ratios was conducted to determine populations significantly enriched or depleted in the mutant tissue. Clusters demonstrating a segregation between mutant and wild-type origin cells were compared to the nearest-related clusters using the Wilcoxon rank-sum test.

### Quantitative PCR

Real-time PCR was performed on cDNA with IQ SYBR Green Supermix (Bio-Rad) and gene-specific primers (Supplementary Methods) and beta-actin (*Actb*) as the reference. The data were analyzed using the ΔΔCt method on the Bio-Rad CFX manager v1.5. Statistical tests used for RT-qPCR analyses are indicated in the corresponding figure legends and source data files. Sox9 expression in Figure 1 was analyzed using the Wilcoxon rank-sum test; other indicated comparisons were analyzed using two-tailed unpaired Student’s t-tests.

## Data Availability

RNA sequence data deposited in GEO (accession number GSE316115). The data generated or analyzed during this study are included in the manuscript and supporting files; source data files have been provided for Total RNA seq for E18.5 embryonic lungs. The Svep1 mouse is available from the Mutant Mouse Resource and Research Center (MMRRC:049943-UCD). The following dataset was generated: LoscertalesM 2026NA-seq analysis of E18.5 mouse embryonic lungs reveals impaired saccular maturation and airway differentiation in Svep1 knockout embryosNCBI Gene Expression OmnibusGSE316115

## Additional files

MDAR checklist
