# Supplementary material for: Svep1 orchestrates distal airway patterning and alveolar differentiation in murine lung development
Source: eLife. 2026 Oct 12;13:RP100443. doi: 10.7554/eLife.100443 (PMC13657628; doi:10.7554/eLife.100443)
Supplement: Peer review history

# Editor report: eLife Assessment

**DOI:** 10.7554/eLife.100443.2.sa0

**Authors:** Anurag Agrawal

**Affiliations:** Ashoka University India

**Keywords:** Important, Solid

This is an **important** piece of work that sheds light on our understanding of early lung development. There is **solid** evidence that there is a key new role for Svep1, which may be acting via FGF9. A more precise understanding of the interactions between Svep1 and FGF9, with a possibility of other ECM factors, would add value.

# Referee report: Reviewer #1 (Public review)

**DOI:** 10.7554/eLife.100443.2.sa1

Summary:

This is an important contribution to the field of molecular embryology of the lung. The authors introduce a novel mesenchymally expressed molecule Svep1. Knocking it out in mice produces a profoundly hypoplastic phenotype which can be rescued in vitro. Svep1 interacts with the FGF signaling complex to control differentiation and expression of smooth muscle in lung mesenchyme, thereby affecting proximal-distal patterning of the airway branches by acting as a putative branch suppressor.

Strengths:

The study shows strong evidence in mouse knockouts, in vitro embryonic lung culture as well as gene expression and in vitro rescue studies that confirm a key role for Svep1. It is a beautiful piece of work and an important contribution to our understanding of early lung branching morphogenesis.

Weaknesses:

Claiming a possible therapeutic role for this gene is a bit far-fetched at the present state of the art.

# Referee report: Reviewer #2 (Public review)

**DOI:** 10.7554/eLife.100443.2.sa2

Summary:

In an effort to elucidate the role of the ECM protein Svep1 in lung development, Foxworth and colleagues have generated a Svep1 mutant (lacking exon 8). Based on their developmental analyses of branching morphogenesis and expression of epithelial, mesenchymal, and mesothelial markers in these mutants, the authors conclude that Svep1 is essential for normal lung growth, morphogenesis, and patterning. They propose that the Svep1 protein regulates, in part, FGF9 signalling. Overall, the paper demonstrates that the ECM is important for lung development and tries to implicate the ECM in the regulation of epithelial-mesenchymal interactions during lung development.

Strengths:

The strengths of this paper are the careful spatiotemporal characterization of (1) lung growth (2) branching morphogenesis (3) epithelial marker expression. The differential perturbation of growth and branching morphogenesis along the D-V axis and the progressive perturbation of branching morphogenesis are both very interesting and noteworthy phenotypes.

Weaknesses:

The weakness of this paper is that it does not present a convincing explanation for how Svep1 regulates any of the phenotypes described. In this regard, a demonstration of a genetic interaction between Svep1 and FGF9 mutants or a careful characterization of a tissue-specific knockdown of Svep1, could be insightful. In addition, a comparison of the phenotype of Svep1 mutants and the phenotypes of other mutants affecting ECM components would be worthwhile.

A minor weakness is that the title of the paper is not fully supported by the data presented. While the defects in the morphogenesis of the distal lung in Svep1 mutants presage a defect in alveolar differentiation, this cannot be formally demonstrated since the animals die soon after birth.

# Author comment: Author response

**DOI:** 10.7554/eLife.100443.2.sa3

**Authors:** Nicole Foxworth, Julie Wells, Sara Ocana-Lopez, S Muller, Pooja Bhayani, James Denegre, Kristina Palmer, Wendy Memishian, Teresa McGee, Steven A Murray, Patricia K Donahoe, Carol J Bult, Maria Loscertales

**Affiliations:** Massachusetts General Hospital Boston United States; Jackson Laboratory Bar Harbor United States; Massachusetts General Hospital Boston United States; Massachusetts General Hospital Boston United States; Massachusetts General Hospital Boston United States; Jackson Laboratory Bar Harbor United States; Jackson Laboratory Bar Harbor United States; Jackson Laboratory Bar Harbor United States; Jackson Laboratory Bar Harbor United States; Jackson Laboratory Bar Harbor United States; Massachusetts General Hospital Boston United States; The Jackson Laboratory Bar Harbor United States; Massachusetts General Hospital Boston United States

**Response to Reviewer #1:**

“Claiming a possible therapeutic role for this gene is a bit far-fetched at the present state of the art”.

We agree that while the therapeutic relevance of Svep1 is not clear at this point, this potential is always something we consider in interpreting our data.

**Response to Reviewer #2:**

a. “The weakness of this paper is that it does not present a convincing explanation for how Svep1 regulates any of the phenotypes described. In this regard, a demonstration of a genetic interaction between Svep1 and FGF9 mutants or a careful characterization of a tissue-specific knockdown of Svep1, could be insightful. In addition, a comparison of the phenotype of Svep1 mutants and the phenotypes of other mutants affecting ECM components would be worthwhile”.

We agree that additional experiments are needed to determine how exactly Svep1 contributes to the phenotypes described. While our preliminary data point to an interaction of Svep1 and Fgf9, we agree that additional data are needed to prove that such interaction is a primary driver of the phenotypes observed.

b. “A minor weakness is that the title of the paper is not fully supported by the data presented. While the defects in the morphogenesis of the distal lung in Svep1 mutants presage a defect in alveolar differentiation, this cannot be formally demonstrated since the animals die soon after birth”

The reviewer is correct that we cannot formally demonstrate this in the current model. The profound defects observed in Svep1 mutants lead to early death, making it challenging to study the full process of alveolarization. However, it is important to note that lung morphogenesis is a continuous process in which earlier developmental phases lay the groundwork for subsequent stages. During the branching phase, the fate of alveolar cell types is established, while the saccular stage serves as a critical foundation for alveolar development, where alveolar cells begin to differentiate. We believe that the significant abnormalities in cellular differentiation observed prior to the bulk of alveolarization indicate likely defects in the later stages of alveolar differentiation. Therefore, while the model limits our ability to directly assess alveolarization, we anticipate that defects in cellular differentiation will continue to manifest beyond the saccular stage in Svep1 mice.
